# Associations between national development indicators and the age profile of people who inject drugs: results from a global systematic review and meta-analysis

**DOI:** 10.1016/S2214-109X(19)30462-0

**Published:** 2019-12-12

**Authors:** Lindsey A Hines, Adam Trickey, Janni Leung, Sarah Larney, Amy Peacock, Louisa Degenhardt, Samantha Colledge, Matthew Hickman, Jason Grebely, Evan B Cunningham, Jack Stone, Kostyantyn Dumchev, Paul Griffiths, Peter Vickerman, Richard P Mattick, Michael Lynskey

**Affiliations:** a Population Health Sciences, Bristol Medical School, University of Bristol, Bristol, UK; b National Drug and Alcohol Research Centre, Sydney, NSW, Australia; c Kirby Institute, University of New South Wales Sydney, Sydney, NSW, Australia; d Ukrainian Institute for Public Health Policy, Kiev, Ukraine; e European Monitoring Centre on Drugs and Drug Addiction, Lisbon, Portugal; f National Addiction Centre, King's College London, London, UK

## Abstract

**Background:**

Globally, an estimated 15·6 million people inject drugs. We aimed to investigate global variation in the age profile of people who inject drugs (PWID), identify country-level factors associated with age of PWID, and assess the association between injecting drug use (IDU) in young people and rates of injecting and sexual risk behaviours at the country level.

**Methods:**

We derived data from a previously published global systematic review done in April, 2016 (and updated in June, 2017) on the percentage of young PWID, duration of IDU, average age of PWID, average age at IDU initiation, and the percentage of PWID reporting sexual and injecting risk behaviours. We also derived national development indicators from World Bank data. We estimated the percentage of young PWID for each country, using a random-effects meta-analysis (DerSimonian-Laird methodology) and generated pooled regional and global estimates for all indicators of IDU in young people. We used univariable and multivariable generalised linear models to test for associations between the age indicators and country urban population growth, youth unemployment percentage, the percentage of PWID who are female, the percentage of the general population aged 15–24 years, Gini coefficient, opioid substitution therapy coverage (per PWID per year), gross domestic product (GDP) per capita (US$1000), and sexual and injecting risk behaviours.

**Findings:**

In the original systematic review, data on age of PWID was reported in 741 studies across 93 countries. Globally, 25·3% (95% uncertainty interval [UI] 19·6–31·8) of PWID were aged 25 years or younger. The highest percentage of young PWID resided in eastern Europe (43·4%, 95% UI 39·4–47·4), and the lowest percentage resided in the Middle East and north Africa (6·9%, 5·1–8·8). At the country level, in multivariable analysis higher GDP was associated with longer median injecting duration (0·11 years per $1000 GDP increase, 95% CI 0·04–0·18; p=0·002), and older median age of PWID (0·13 years per $1000 increase, 0·06–0·20; p<0·0001). Urban population growth was associated with higher age at IDU initiation (1·40 years per annual percentage change, 0·41–2·40). No associations were identified between indicators of IDU in young people and youth unemployment, Gini coefficient, or opioid substitution therapy coverage provision at the country level. No associations were identified between injecting and sexual risk behaviours and age of PWID.

**Interpretation:**

Variation in the age profile of PWID was associated with GDP and urbanisation. Regions with the highest prevalence of young PWID (aged ≤25 years) had low coverage of interventions to prevent the spread of blood-borne viruses. Data quality highlights the need for improvements in monitoring of PWID populations.

**Funding:**

Australian National Drug and Alcohol Research Centre, Australian National Health and Medical Research Council, Open Society Foundation, WHO, the Global Fund, UNAIDS, National Institute for Health Research Health Protection Research Unit for Evaluation of Interventions, Wellcome Trust.

## Introduction

Globally, an estimated 15·6 million people aged 15–64 years inject drugs (95% uncertainty interval [UI] 10·2–23·7).^1^ Injecting drug use (IDU) is of high public health importance because of the associated elevated risks of overdose, drug dependence, and blood-borne virus transmission.^2^ Subsequently, IDU is an important contributor to the global burden of disease.^3^

The initiation of IDU among young people is of great concern. Younger age of onset of IDU is associated with overdose,^4^ faster progression to regular heroin use,^5^ and lower treatment uptake.^6^ Young people who inject drugs (PWID) have been found to be more likely to share needles and syringes^7^ and can be at high risk for engagement in sexual behaviour, facilitating the spread of blood-borne viruses.^8^ Preventing the uptake of IDU among young people presents an opportunity to reduce the spread of these viruses to new generations and consequently reduce the associated health-care burden.

**Research in context**

**Evidence before this study**

In our 2017 systematic review, we identified wide country-level variation in the percentage of young people who inject drugs (PWID), which is a concern since young age of onset of injecting drug use (IDU) is associated with greater risk of overdose (regardless of duration of drug use), faster progression to regular heroin use, and lower uptake of drug treatment. To date, research into the predictors of the age profile of PWID has largely focused on individual-level factors such as childhood abuse and employment. Little is known about the country-level characteristics that might contribute to higher numbers of young PWID.

**Added value of this study**

In this study, we assessed IDU among younger people within the context of global development and identified substantial variation in the percentage of young PWID. The estimated global percentage of PWID aged 25 years or younger is 25·3% (around 3·9 million young PWID), but between countries these estimated percentages range from 5·1% to 53·8%. This study is the first to explore country-level factors that might contribute to this variation, and is the first to assess associations between IDU in young people and injecting and sexual risk behaviours at the country level. Lower gross domestic product (GDP) per capita was associated with a shorter median duration of injecting, and a lower median age of PWID. Lower urbanisation growth rates were associated with a younger age of onset of IDU. The results provide a compelling argument that growth and development might have implications for periods of epidemic drug use, and for necessary provision of measures to prevent the spread of blood-borne viruses.

**Implications of all the available evidence**

Assessment of the global variation in IDU among young people has identified countries in which youth IDU urgently needs to be addressed, with this group accounting for up to half of the PWID population in some countries. Many of the regions with the highest percentages of young PWID had the lowest provision of harm reduction to prevent the spread of HIV and hepatitis C. Nationally, GDP and urban population growth were associated with indicators of youth injecting and consequently, greater consideration of IDU within the context of development goals is warranted.

Epidemiological data on PWID shows wide between-country variation in the age profile of PWID, with the proportion of young PWID ranging from 6·7% in Kyrgyzstan and Ghana to 71% in Turkey.^1^ To date, research on the correlates of IDU has largely focused on individual-level factors.^9^, ^10^ Less is known about the national-level characteristics that might contribute to differences in the age profile of PWID.

Adolescent health faces challenges in the context of modern development. The largest proportion of young people (aged 15–24 years) reside in Africa, Asia, and Latin America;^11^ regions that comprise the highest numbers of low-income and middle-income countries (LMICs), and are typically undergoing urbanisation and its associated challenges.^12^ Addressing mental health needs of young people has been identified as a global challenge,^13^ but despite illicit drug use contributing to the global burden of disease among young people,^14^ to what extent IDU varies among young people globally remains unknown.

By combining epidemiological data extracted during our previous global systematic review^1^ with national socioeconomic data from the World Bank,^15^, ^16^ we aimed to assess between-country variation in PWID age indicators (percentage of PWID aged ≤25 years, median age of PWID, age of onset of injecting, and duration of IDU); the association between urban population growth, youth unemployment, per capita gross domestic product (GDP), Gini coefficient, opioid substitution therapy, and indicators of the age of the IDU population at the country level; and the association between youth IDU and rates of injecting and sexual risk behaviour at the country level.

## Methods

### Search strategy and selection criteria

Country-level data on the percentage of young PWID, duration of IDU, age of IDU onset, and average age of IDU were derived from our previous multistage global systematic review on the prevalence of IDU and characteristics of PWID.^1^ Full methods of our previous systematic review have been published elsewhere.^1^ Briefly, we searched electronic peer-reviewed literature databases (MEDLINE, Embase, and PsycINFO), grey literature, online databases,^17^ and key documents published by relevant international agencies and experts. Searches were restricted to studies published since Jan 1, 2008 (since the aim of the original study was to update estimates produced in 2008). Searches of the grey literature were done between April and June, 2016, and updated between May and June, 2017, and searches of peer-reviewed literature were done in June, 2017. Identified experts were emailed to request additional information. Search terms are provided in the published paper.^1^ No language restrictions were used, and non-English languages were read by the research team or via translation programs.

### Screening and selection

Studies were screened on the basis of pre-specified decision rules and study quality assessment, with higher quality studies selected over lower quality studies.^1^ Initial title and abstract screening was done independently by one reviewer, with a random 10% of studies checked by a second person (LD, SL, or AP). Full-text review was done independently by two authors (LD, SL, or AP), with any discrepancies resolved by consensus or by a third reviewer (MH) for searches that yielded fewer than 30 records; a consensus was reached in all instances. We extracted data at all levels reported in the study, including city, subnational, and country. Data were then checked for accuracy against the original source by one of three authors (LD, SL, or AP). The review was reported in accordance with PRISMA^18^ and GATHER^19^ guidelines,^1^ and the protocols were registered on PROSPERO, numbers CRD42016052858 and CRD42016052853.^17^, ^20^ The national development indicators were obtained from the World Bank Data Catalog.^15^

### Data extraction

Full details of all outcomes are in the appendix (pp 2–4). Studies recorded the percentages of PWID (at time of study data collection) in different age categories for current injectors. To ensure our analysis focused on young people, to estimate the percentage of young PWID, we excluded data from studies in which the youngest age group included adults aged older than 25 years.^21^

We extracted data on median duration of IDU and average age of PWID at the time of study data collection where reported, and data on average age of IDU onset at time of study data collection, if directly reported.

For age of PWID and age of onset of IDU, we used a median (range) where available and a mean (range) where a median was not available but a mean was; hence, we refer to these as averages throughout, rather than specifically as means and medians. Duration of IDU is presented as a median (range).

To assess whether measures of youth IDU were associated with sociodemographic and wealth indicators, we derived data on the following development indicators for 2015 from The World Bank Catalog and the UN:^11^, ^15^, ^16^ GDP per capita (US$ thousand), youth unemployment (proportion of labour force aged 15–24 years), urban population growth (annual percentage change in the population residing in urban areas), Gini coefficient (measuring inequality on a scale from 0–100, whereby a lower score indicates higher equality), and the proportion of the general population aged 15–24 years.^16^ We obtained data from previous meta-analyses for measures of opioid substitution therapy coverage^22^ (per PWID per year) and the percentage of female PWID at the country level.^23^

We also estimated the percentage of PWID who had recently engaged in injecting risk behaviour (predominantly receptive needle sharing, typically in the past month) and the percentage of PWID who had recently engaged in sexual risk behaviour (predominantly no or inconsistent condom use with casual partner, typically within the past month).

### Data analysis

All analyses were done using Stata (version 15.0), with the exception of mapping, which was done in Tableau.

We estimated the percentage of young PWID aged 25 years and under for each country, pooled using a random-effects meta-analysis (DerSimonian-Laird methodology) via the Metaprop command in Stata 15.0. The number of people included in the young age group and the overall sample size for each study were specified, using the Freeman-Tukey double arcsine transformation to stabilise the variances. We used the *I*^2^ statistic to assess heterogeneity between studies for the percentage of young PWID, for each country.^24^

The tabulations for the average age of PWID, average age of onset of injecting, and average duration of injecting were produced by weighting each study according to sample size and creating a mean (range) for each country. The percentage of PWID who were young, and the median years of injecting among PWID, were mapped for each country.

Regional and global estimates for all indicators of youth IDU were pooled by weighting the size of the PWID population in the countries within each region.

We used generalised linear models to test for associations using the percentage of young PWID (converted to a proportion and logit transformed: log[p/(1-p)]), average age of PWID, average age of onset of injecting, and median duration of injecting in the country as the dependent variables, and urban population growth, youth unemployment, the percentage of PWID who were women, the percentage of the general population aged 15–24 years, Gini coefficient, opioid substitution therapy coverage (per PWID per year), and GDP per capita (US$ thousand) as independent variables. Analyses were done at the country level because our aim was to analyse the variation in the percentage of young PWID between countries, rather than between studies. All independent variables associated with the dependent variables in the univariable analyses (p<0·05) were entered in multivariable analyses. This method was repeated using the percentage of PWID reporting injecting risk and the percentage of PWID reporting sexual risk behaviour (both logit transformed) as dependent variables. Median age, duration of injecting, age of onset of injecting, and the percentage of young PWID were modelled separately as the independent variables. Independent variables associated with the dependent variables (p<0·05) in the univariable analyses were entered in multivariable analyses. Estimates lacking information on the origin of the source study were excluded. A conservative Bonferroni correction of 0·008 (ie, 0·05/6) was used as the p value for the multivariable analyses to account for multiple hypothesis testing in the six multivariable models.

We used sensitivity analyses to investigate the effect of defining young PWID with different age cutoffs (appendix pp 1–3).

### Role of the funding source

The funders of the study had no role in the study design, data collection, data analysis, data interpretation, or writing of the report. The corresponding author had full access to all of the data in the study and had final responsibility for the decision to submit for publication.

## Results

**Figure 1:**
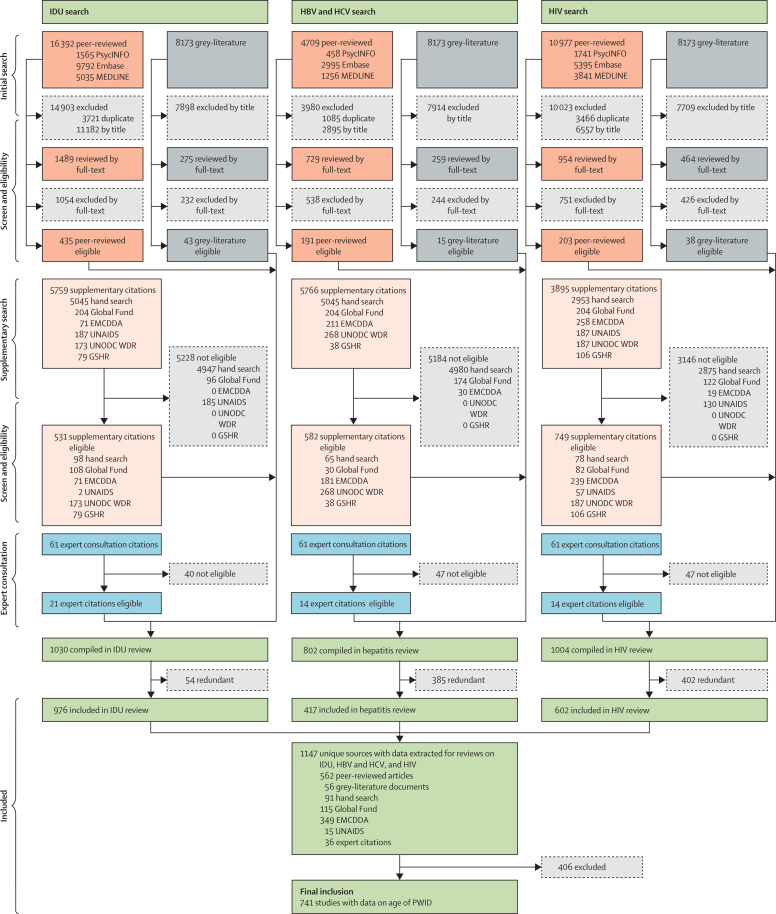
Flowchart of study inclusion criteria Adapted from Degenhardt and colleagues.^1^ EMCDDA=European Monitoring Centre on Drugs and Drug Addiction. GSHR=HRI's Global State of Harm Reduction. HBV=hepatitis B virus. HCV=hepatitis C virus. IDU=injecting drug use. PWID=people who inject drugs. UNODC WDR=UN Office on Drugs and Crime's World Drug Report.

The systematic search^1^ yielded 55 671 studies, of which 1147 studies met inclusion criteria (figure 1). Data on age of PWID was reported in 741 studies across 93 countries (appendix pp 5–46), accounting for 79% of the global population. All study variables and country-level *I*^2^ values are shown in the appendix (pp 5–7).

Globally, 25·3% (95% UI 19·6–31·8) of PWID were young (aged ≤25 years), equating to approximately 3·9 million (3·1–5·0) young people. The average age of PWID was 32·5 years (range 18–55), and the average age of onset of IDU was 23·3 years (13–39). The weighted mean of the median durations of IDU was 9·7 years (1–25) across studies.

**Figure 2:**
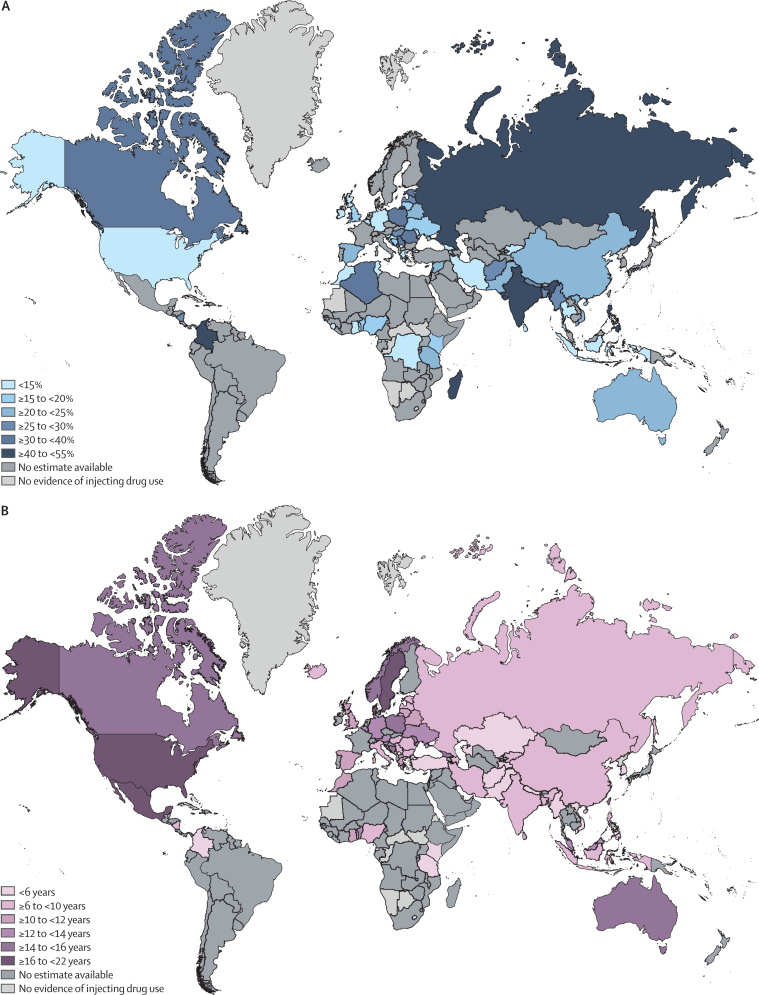

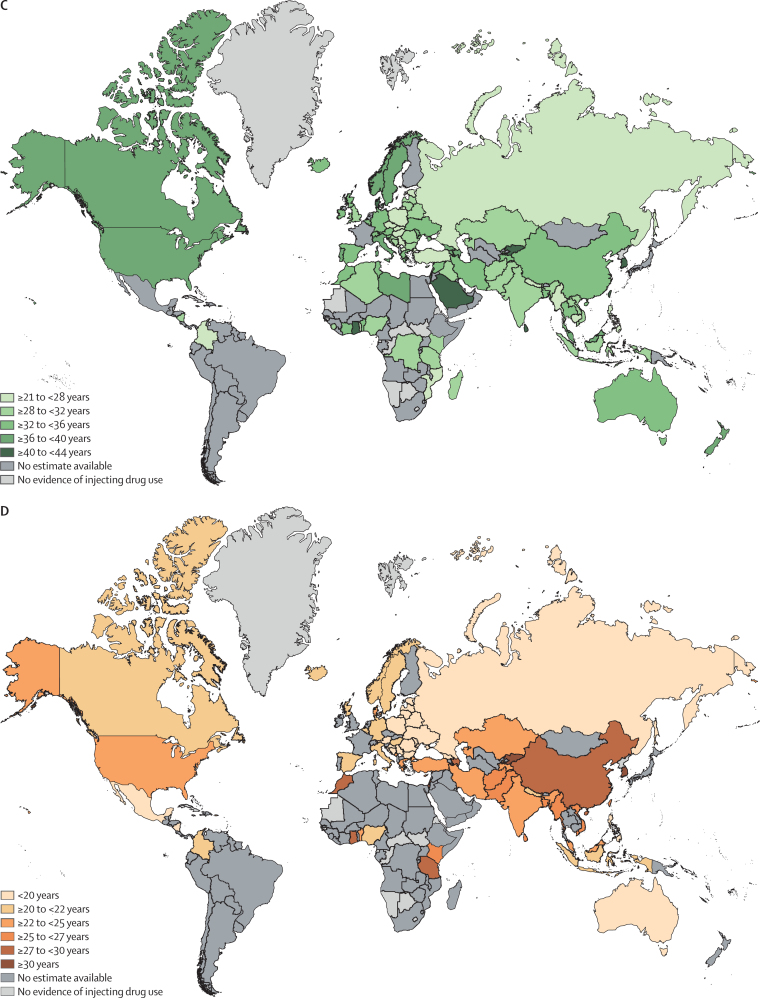
Estimated percentage of young PWID (A), median duration of injecting drug use among PWID (B), average age of PWID population (C), and average age of onset of injecting among PWID (D), by country PWID=people who inject drugs.

Large variations were identified in the estimated percentage of young PWID by region and by country (table 1; figure 2A). The highest percentage of young PWID resided in eastern Europe (43·4%, 95% UI 39·4–47·4) and the lowest in the Middle East and north Africa (6·9%, 5·1–8·8). The shortest regional duration of IDU was estimated in sub-Saharan Africa (4·6 years, range 3–17), and the longest duration in North America (16·0 years, 4–21; table 1; figure 2B). The youngest median age of PWID in a region was estimated to be in eastern Europe (27·9 years, range 19–43) and the oldest was in North America (37·6 years, 28–55; table 1; figure 2C). The youngest age of IDU onset was estimated to be in Australasia (19·1 years, range 17–20) and the oldest was in the Middle East and north Africa (28·9 years, range 20–29; table 1; figure 2D).

**Table 1:** Age and injecting history parameters by country and region

		Estimated number of PWID, n (95% UI)*	**Young PWID, % (95% CI)**	**Average age of PWID, years (range)**	**Average age of onset of injecting, years (range)**	**Median duration of injecting, years (range)**
Global		15 648 000 (10 219 000–23 737 500)	25·3% (19·6–31·8)	32·5 (18–55)	23·3 (13–39)	9·7 (1–25)
Eastern Europe						
	Overall	3 002000 (1 653 500–5 008 000)	43·4% (39·4–47·4)	27·9 (19–43)	19·4 (15–29)	8·7 (2–20)
	Armenia	13 000 (9000–29 000)	..	38·5 (34–43)	..	..
	Azerbaijan	43 500 (34 500–52 000)	..	36·0 (33–40)	27·3 (25–29)	8·8 (5–12)
	Belarus	40 500 (15 000–66 000)	20·1% (10·5–31·9)	30·8 (26–35)	19·0 (17–20)	10·9 (8–13)
	Bosnia and Herzegovina	..	16·6% (7·9–27·6)	31·0 (25–33)	15·3 (15–16)	15·0 (9–17)
	Bulgaria	18 500 (15 000–22 500)	..	29·4 (28–30)	19·0 (19–19)	9·0 (9–9)
	Czech Republic	47 000 (44 500–49 000)	..	27·5 (27–31)	..	..
	Estonia	8500 (6500–16 000)	35·4% (26·2–45·2)	27·6 (23–30)	19·6 (18–22)	8·1 (5–11)
	Georgia	115 000 (13 000–217 000)	12·7% (9·5–16·3)	36·9 (32–40)	19·4 (18–26)	14·1 (5–20)
	Hungary	4000 (2000–6000)	26·8% (21·7–32·3)	26·7 (23–33)	21·3 (20–22)	9·6 (8–11)
	Latvia	14 000 (11 000–18 000)	28·2% (24·9–31·6)	28·6 (28–30)	19·4 (19–20)	9·1 (8–10)
	Lithuania	5000 (2500–8000)	18·0% (14·4–22·1)	29·4 (29–30)	19·0 (19–19)	10·0 (10–10)
	Moldova	12 000 (7500–16 500)	13·4% (6·5–22·3)	32·2 (27–36)	19·5 (17–21)	12·7 (10–15)
	Poland	..	37·5% (18·7–58·6)	27·8 (21–35)	18·0 (18–18)	14·4 (13–16)
	Romania	81 500 (60 500–110 000)	31·8% (27·7–36·0)	28·7 (27–31)	18·5 (16–20)	9·8 (8·1–11)
	Russia	1 881 000 (1 028 500–3 114 000)	52·1% (48·3–55·9)	26·2 (19–33)	19·4 (17–21)	7·6 (2–10)
	Slovakia	20 000 (14 500–36 000)	..	26·5 (25–29)	..	..
	Ukraine	319 500 (172 000–590 500)	19·8% (15·0–25·0)	32·1 (29–35)	18·5 (18–23)	12·2 (6–16)
Western Europe						
	Overall	1 009 500 (686 500–1 386 500)	14·1% (10·2–18·7)	34·0 (21–48)	20·5 (18–26)	10·7 (5–22)
	Albania	..	..	..	..	..
	Andorra	..	..	..	..	..
	Austria	185 00 (12 500–24 500)	..	32·0 (32–32)	19·0 (19–19)	13·0 (13–13)
	Belgium	26 000 (18 500–37 000)	14·4% (11·3–17·8)	35·4 (34–37)	21·7 (21–22)	13·3 (13–14)
	Croatia	6500 (5000–8500)	14·4% (7·8–22·6)	31·3 (26–37)	19·5 (18–25)	13·5 (5–19)
	Denmark	16 500 (13 000–19 000)	..	43·5 (37–45)	23·8 (18–25)	18·2 (18–19)
	England	210 500 (196 500–225 000)	19·5% (16·4–22·8)	28·6 (22–36)	..	10·0 (10–10)
	Finland	17 000 (15 000–25 000)	..	..	..	..
	France	82 000 (66 500–97 000)	..	..	..	..
	Germany	131 500 (14 000–249 500)	6·6% (3·6–10·5)	35·8 (28–41)	21·4 (19–23)	13·9 (8–18)
	Greece	5000 (4000–6000)	12·8% (6·6–20·7)	32·6 (27–36)	23·8 (21–24)	11·7 (8–12)
	Iceland	..	..	33·0 (33–33)	20·0 (20–20)	7·0 (7–7)
	Ireland	8500 (6500–10 500)	8·9% (6·1–12·4)	32·5 (30–33)	..	..
	Italy	341 500 (233 500–467 500)	..	36·5 (29–46)	20·0 (20–20)	9·0 (9–9)
	Luxembourg	2000 (1500–2500)	..	..	..	..
	Malta	..	..	..	..	..
	Monaco	..	..	..	..	..
	Montenegro	..	39·0% (33·7–44·5)	27·0 (25–29)	23·0 (23–23)	6·0 (6–6)
	Netherlands	3500 (2500–4500)	..	..	..	..
	North Macedonia	..	..	27·5 (21–36)	..	..
	Northern Ireland	..	..	..	..	..
	Norway	8500 (7000–10 000)	..	36·9 (33–38)	20·0 (20–20)	14·0 (14–14)
	Portugal	16 000 (13 500–17 500)	..	38·0 (38–38)	..	..
	San Marino	..	..	..	..	..
	Scotland	16 000 (13 500–17 500)	12·1% (7·9–17·2)	34·5 (26–37)	21·4 (20–22)	11·5 (7–13)
	Serbia	29 000 (24 000–34 500)	30·7% (15·7–48·2)	27·9 (23–31)	19·6 (18–20)	8·8 (5–11)
	Slovenia	6000 (4000–7500)	10·8% (6·9–15·9)	33·0 (33–33)	..	..
	Spain	10 500 (3500–17 500)	20·5% (4·9–43·0)	35·7 (27–41)	21·0 (21–21)	11·2 (7–15)
	Sweden	8000 (2000–38 500)	..	37·1 (30–48)	20·8 (19–26)	21·0 (14–22)
	Switzerland	13 500 (11 000–16 000)	..	35·0 (35–35)	..	..
	Wales	..	27·4% (24·2–30·9)	30·0 (30–30)	..	9·0 (9–9)
East and southeast Asia						
	Overall	3 989 000 (3 041 000–4 955 000)	22·0% (16·5–28·0)	32·7 (21–53)	26·4 (13–39)	7·4 (1–25)
	Brunei	..	..	..	..	..
	Cambodia	10 500 (9500–22 500)	..	25·5 (23–29)	..	..
	China	2 564 000 (1 964 000–3 164 000)	22·3% (17·2–27·8)	32·9 (26–53)	27·3 (24·5–36)	7·1 (1·5–25)
	Hong Kong	..	..	49·0 (49–49)	32·0 (32–32)	17·0 (17–17)
	Indonesia	190 500 (156 000–225 000)	14·1% (7·0–23·2)	29·4 (22–33)	20·2 (13–22)	7·1 (4–13)
	Japan	368 500 (281 500–459 000)	..	..	..	..
	Laos	..	..	30·0 (30–30)	..	..
	Malaysia	281 500 (233 500–330 000)	..	38·0 (37–39)	24·0 (24–24)	13·9 (13–15)
	Mongolia	..	..	..	..	..
	Myanmar	173 500 (115 500–235 000)	25·2% (21·3–29·2)	27·2 (24–33)	26·0 (22–31)	3·4 (1–11)
	Philippines	25 500 (19 000–32 000)	44·3% (16·2–74·6)	25·3 (21–30)	18·5 (18–19)	6·8 (3–11)
	South Korea	..	..	41·2 (39–42)	36·5 (29–39)	4·8 (3–10)
	Singapore	..	..	43·0 (43–43)	..	..
	Taiwan	..	..	40·8 (37–45)	26·4 (26–27)	15·5 (14–18)
	Thailand	51 500 (16 000–87 000)	9·7% (3·1–19·4)	32·2 (27–42)	..	..
	Timor Leste	500 (500–500)	..	..	..	..
	Vietnam	161 000 (123 000–200 500)	22·8% (15·6–30·8)	31·7 (22–38)	25·9 (21–32)	5·8 (4–8)
South Asia						
	Overall	1 023 500 (783 500–1 263 000)	26·4% (21·5–31·5)	30·3 (20–44)	25·2 (16–35)	5·7 (1–21)
	Afghanistan	139 000 (88 000–190 500)	25·3% (19·8–31·1)	28·3 (21–32)	26·2 (24–28·5)	2·5 (1–4)
	Bangladesh	68 500 (63 500–74 000)	28·7% (26·3–31·1)	32·6 (27–42)	26·5 (22–35)	6·0 (1–9)
	Bhutan	..	..	..	..	..
	India	197 000 (127 500–267 000)	42·8% (34·5–51·3)	29·4 (21–39)	22·0 (16–31)	7·2 (2–21)
	Iran	158 000 (107 000–209 000)	14·9% (11·6–18·4)	32·7 (27–44)	24·3 (19–29)	8·2 (3–19)
	Maldives	1500 (500–2500)	39·1% (33·4–45·0)	25·5 (25–26)	22·0 (22–22)	3·5 (3–4)
	Nepal	35 000 (33 500–37 000)	27·2% (15·0–41·4)	24·2 (20–33)	20·7 (19–23)	5·2 (3–12)
	Pakistan	423 000 (363 000–482 500)	22·9% (19·4–26·6)	30·5 (28–34)	26·9 (22–29)	5·1 (5–8)
	Sri Lanka	500 (500–500)	8·6% (5·8–12·2)	40·0 (40–40)	26·0 (26–26)	11·0 (11–11)
Central Asia						
	Overall	281 500 (189 500–416 500)	7·0% (5·4–8·8)	32·5 (30–46)	24·8 (23–32)	5·7 (4·6–7)
	Kazakhstan	112 500 (75 500–166 000)	..	30·0 (30–30)	23·0 (23–23)	5·0 (5–5)
	Kyrgyzstan	28 500 (19 000–42 000)	7·0% (5·4–8·8)	41·0 (36–46)	30·5 (28–32)	6·3 (5–7)
	Tajikistan	235 000 (16 000–34 500)	..	32·6 (31–38)	24·9 (24–26)	5·9 (4·6–7)
	Turkmenistan	..	..	..	..	..
	Uzbekistan	94 000 (63 000–140 000)	..	..	..	..
Caribbean						
	Overall	79 500 (53 000–118 000)	..	NE (36–42)	NE (20–24)	NE (10–17)
	The Bahamas	..	..	..	..	..
	Bermuda	..	..	..	..	..
	Dominican Republic	..	..	..	..	..
	Haiti	..	..	..	..	..
	Jamaica	..	..	..	..	..
	Puerto Rico	..	..	40·5 (36–42)	21·2 (20–24)	12·9 (10–17)
Latin America						
	Overall	1 823 000 (1 392 000–2 380 000)	..	NE (18–37)	19·3 (17–21)	16·1 (3–18)
	Argentina	80 500 (79 000–82 500)	..	..	..	..
	Bolivia	..	..	..	..	..
	Brazil	962 000 (734 500–1 256 000)	..	..	..	..
	Chile	47 000 (36 000–61 500)	..	..	..	..
	Colombia	..	53·8% (46·4–61·1)	21·6 (18–25)	20·0 (20–20)	3·0 (3–3)
	Costa Rica	..	..	..	..	..
	Ecuador	..	..	..	..	..
	El Salvador	..	..	..	..	..
	Guatemala	..	..	..	..	..
	Guyana	..	..	..	..	..
	Honduras	..	..	..	..	..
	Mexico	150 500 (100 500–209 500)	..	..	19·3 (17–21)	16·1 (15–18)
	Nicaragua	..	29·3 (16·1–45·5)	30·0 (30–30)	18·0 (18–18)	6·0 (6–6)
	Panama	..	..	..	..	..
	Paraguay	..	..	..	..	..
	Peru	..	..	..	..	..
	Suriname	..	..	..	..	..
	Uruguay	6500 (2000–9000)	..	..	..	..
	Venezuela	..	..	..	..	..
North America						
	Overall	2 557 000 (1 498 500–4 428 000)	13·3% (6·0–22·6)	37·6 (28–55)	22·3 (18–29)	16·0 (4–21)
	Canada	308 000 (262 000–354 500)	33·5% (16·1–53·6)	36·9 (30–48)	21·9 (18–26)	14·3 (4–20)
	USA	2 248 500 (1 236 500–4 074 000)	10·5% (4·6–18·3)	37·7 (28–55)	22·4 (19–29)	16·2 (5–21)
Pacific Island States and Territories						
	Overall	22 500 (15 000–33 500)	..	..	..	..
	American Samoa	..	..	..	..	..
	Federated States of Micronesia	..	..	..	..	..
	Fiji	..	..	..	..	..
	French Polynesia	..	..	..	..	..
	Guam	..	..	..	..	..
	Kiribati	..	..	..	..	..
	Marshall Islands	..	..	..	..	..
	New Caledonia	..	..	..	..	..
	Northern Mariana Islands	..	..	..	..	..
	Palau	..	..	..	..	..
	Papua New Guinea	..	..	..	..	..
	Samoa	..	..	..	..	..
	Solomon Islands	..	..	..	..	..
	Tonga	..	..	..	..	..
	Vanuatu	..	..	..	..	..
Australasia						
	Overall	115 500 (83 000–148 000)	22·5% (9·8–38·5)	35·6 (25–42)	19·1 (17–20)	15·4 (8–22)
	Australia	93 000 (68 000–93 000)	22·5% (9·8–38·5)	34·7 (25–42)	19·1 (17–20)	15·4 (8–22)
	New Zealand	22 500 (15 000–30 000)	..	39·2 (33–42)	..	..
Sub-Saharan Africa						
	Overall	1 378 000 (645 500–3 080 000)	20·7% (10·5–32·9)	29·4 (21–42)	28·2 (20–36)	4·6 (3–17)
	Angola	..	..	..	..	..
	Benin	..	17·7% (14·5–21·2)	34·3 (32–35)	22·0 (22–22)	13·0 (13–13)
	Burkina Faso	..	..	..	..	..
	Burundi	..	..	..	..	..
	Cameroon	..	..	..	..	..
	Cape Verde	..	..	..	..	..
	Chad	..	..	..	..	..
	Democratic Republic of the Congo	3500 (0–158 000)	5·1% (1·7–11·5)	31·0 (31–31)	..	..
	Côte d'Ivoire	500 (500–1000)	..	35·0 (35–35)	..	..
	Djibouti	..	..	..	..	..
	Ethiopia	..	..	..	..	..
	Gabon	..	..	..	..	..
	The Gambia	..	..	..	..	..
	Ghana	..	6·7% (2·5–13·9)	42·0 (42–42)	27·0 (27–27)	10·0 (10–10)
	Guinea	..	..	..	..	..
	Kenya	30 500 (9000–52 000)	15·0% (11·4–18·9)	30·1 (26–32)	26·6 (23–33)	5·3 (4–6)
	Liberia	..	..	..	..	..
	Madagascar	15 500 (3000–79 500)	45·3% (10·8–82·6)	28·8 (21–31)	..	..
	Malawi	..	..	..	..	..
	Mali	..	..	..	..	..
	Mauritius	7000 (3500–14 000)	10·6% (8·0–13·6)	34·5 (31–38)	20·0 (20–20)	14·0 (11–17)
	Mozambique	29 000 (0–59 000)	..	22·0 (22–22)	..	..
	Namibia	..	..	..	..	..
	Niger	..	..	..	..	..
	Nigeria	..	17·2% (6·9–31·0)	30·7 (30–40)	20·0 (20–20)	8·0 (8–8)
	Rwanda	2000 (500–4500)	..	..	..	..
	Senegal	..	..	..	..	..
	Seychelles	1500 (1000–2500)	28·0% (23·4–33·1)	28·0 (28–28)	..	..
	Sierra Leone	1500 (1000–1500)	8·1% (5·1–12·1)	29·0 (29–29)	..	..
	Somalia	..	..	..	..	..
	South Africa	76 000 (21 500–268 000)	..	..	..	..
	eSwatini	..	..	..	..	..
	Togo	2500 (500–19 500)	9·7% (6·4–13·8)	27·0 (27–27)	..	..
	Uganda	..	..	..	..	..
	Tanzania	343 000 (200 000–486 000)	20·6% (10·6–32·7)	29·9 (27–35)	28·5 (20–36)	4·3 (3–9)
	Zambia	..	..	..	..	..
	Zimbabwe	..	..	..	..	..
Middle East and north Africa						
	Overall	349 500 (177 500–521 500)	6·9% (5·1–8·8)	33·5 (27–43)	28·9 (20–29)	10·0 (3–14)
	Algeria	..	36·8% (27·2–47·4)	30·0 (30–30)	..	..
	Bahrain	..	..	..	..	..
	Cyprus	500 (500–1000)	..	31·5 (27–32)	22·7 (20–23)	8·8 (7–9)
	Egypt	..	..	..	..	..
	Iraq	..	..	28·0 (28–28)	..	..
	Israel	..	..	43·0 (43–43)	29·0 (29–29)	14·0 (14–14)
	Jordan	..	..	..	..	..
	Kuwait	..	..	..	..	..
	Lebanon	..	17·3% (9·8–27·3)	29·5 (29–30)	..	..
	Libya	2000 (1000–3000)	..	39·0 (39–39)	..	..
	Morocco	30 500 (15 500–45 500)	6·9% (5·1–8·8)	33·2 (31–39)	29·0 (29–29)	10·0 (10–10)
	Occupied Palestinian territory	..	..	40·6 (39–43)	29·0 (29–29)	14·0 (14–14)
	Oman	..	..	..	..	..
	Qatar	..	..	..	..	..
	Saudi Arabia	..	..	40·0 (40–40)	..	..
	Sudan	..	..	..	..	..
	Syria	..	24·4% (20·2–28·9)	32·0 (32–32)	..	..
	Tunisia	..	14·6% (12·2–17·2)	34·6 (33–36)	..	..
	Turkey	..	..	27·2 (27–32)	23·2 (23–28)	4·0 (3–4)
	United Arab Emirates	..	..	..	..	..
	Yemen	..	..	..	..	..

For age of PWID and age of onset of IDU, we used a median (range) where available and a mean (range) where a median was not available but a mean was; hence, we refer to these as averages throughout. Data on injecting drug use were not available for Antigua and Barbuda, Barbados, Belize, Botswana, Central African Republic, Comoros, Cuba, Dominica, Equatorial Guinea, Eritrea, Greenland, Grenada, Guinea-Bissau, Lesotho, Liechtenstein, Mauritania, Namibia, Nauru, North Korea, Republic of the Congo, Saint Kitts and Nevis, Saint Lucia, São Tomé and Principe, Saint Vincent and the Grenadines, South Sudan, Trinidad and Tobago, and Tuvalu and thus these countries are not listed. Young PWID were defined as individuals aged younger than 25 years where possible; some studies used slightly different age groupings. Full details of all included studies are in the appendix (pp 5–46). .. indicates no data available. NE=not estimable. PWID=people who inject drugs. UI=uncertainty interval.

* Data obtained from Degenhardt and colleagues.^1^

**Figure 3:**
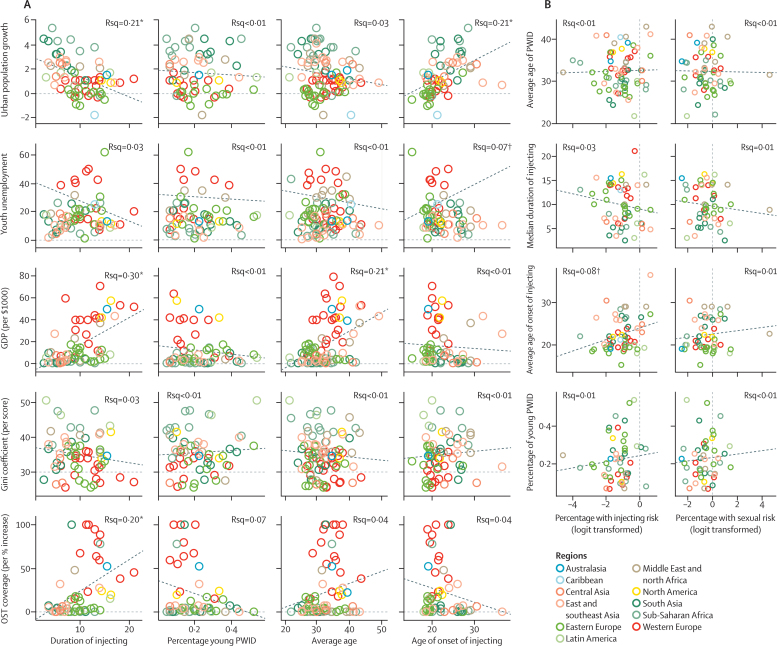
Country-level predictors of indicators of youth injecting drug use (A) and the association between injecting and sexual risk variables and age and injecting history (B) The variables on the percentage of PWID with injecting risk and sexual risk were logit transformed. Rsq gives the R^2^ value that is proportional to how much of the variability in the dependent variable (eg, duration of injecting) is explained by the independent variable (eg, GDP). GDP=gross domestic product. OST=opioid substitution therapy. *p<0·001. †p<0·05.

Higher country-level urban population growth was associated with increased age of IDU onset and a shorter duration of IDU in univariable analyses. However, this association was only maintained in multivariable analysis for age of IDU onset (Table 2, Table 3; figure 3A). No associations were identified between urban population growth and indicators of youth IDU in multivariable analysis.

**Table 2:** Country-level linear regression analyses of the predictors of duration of injecting among PWID and the age profile of PWID (logit transformed)

	**Duration of injecting**					**Percentage of PWID who are young**				
	Univariable analysis (n=68)			Multivariable analysis (n=53)		Univariable analysis (n=60)			Multivariable analysis (n=NA)	
	n	Coefficient (95% CI)	p value	Coefficient (95% CI)	p value	n	Coefficient (95% CI)	p value	Coefficient (95% CI)	p value
Urban population growth	66	−1·25 (−1·86 to −0·63)	0·0001	−1·06 (−2·05 to −0·07)	0·036	60	−0·04 (−0·15 to 0·07)	0·486	..	..
Youth unemployment	66	0·06 (−0·02 to 0·14)	0·165	..	..	59	0·00 (−0·01 to 0·02)	0·957	..	..
GDP (per US$1000 increase)	65	0·12 (0·07 to 0·17)	<0·0001	0·11 (0·04 to 0·18)	0·002	59	−0·01 (−0·02 to 0·01)	0·329	..	..
Gini coefficient (per score increase)	65	−0·11 (−0·28 to 0·06)	0·206	..	..	59	0·00 (−0·03 to 0·04)	0·881	..	..
Opioid substitution therapy coverage (per % increase in coverage)	55	0·06 (0·03 to 0·09)	0·001	0·02 (−0·02 to 0·05)	0·320	51	−0·01 (−0·01 to 0·00)	0·121	..	..
Percentage of PWID who are women	63	0·14 (0·05 to 0·24)	0·004	−0·01 (−0·13 to 0·12)	0·914	53	0·00 (−0·02 to 0·02)	0·843	..	..
Proportion of the general population aged 15–24 years	64	−0·59 (−0·86 to −0·31)	<0·0001	0·02 (−0·40 to 0·55)	0·928	57	−0·01 (−0·06 to 0·06)	0·704	..	..

n refers to number of countries. The percentage of PWID who are young variable was logit transformed. GDP=gross domestic product. NA=not applicable. PWID=people who inject drugs.

**Table 3:** Country-level linear regression analyses of the predictors of the average age of PWID and average age of onset of injecting

	**Average age of PWID**						**Average age of onset of injecting**			
	Univariable analysis (n=94)			Multivariable analysis (n=79)		Univariable analysis (n=66)			Multivariable analysis (n=61)	
	n	Coefficient (95% CI)	p value	Coefficient (95% CI)	p value	n	Coefficient (95% CI)	p value	Coefficient (95% CI)	p value
Urban population growth	92	−0·52 (−1·18 to 0·15)	0·129	..	..	64	1·20 (0·62 to 1·78)	0·0001	1·40 (0·41 to 2·40)	0·007
Youth unemployment	91	−0·01 (−0·09 to 0·08)	0·887	..	..	64	−0·09 (−0·16 to −0·01)	0·032	−0·05 (−0·14 to 0·04)	0·257
GDP (per US$1000 increase)	89	0·12 (0·07 to 0·17)	<0·0001	0·13 (0·06 to 0·20)	0·0004	63	−0·01 (−0·07 to 0·04)	0·631	..	..
Gini coefficient (per score increase)	87	−0·06 (−0·23 to 0·11)	0·496	..	..	63	0·05 (−0·12 to 0·22)	0·539	..	..
Opioid substitution therapy coverage (per % increase in coverage)	76	0·03 (−0·00 to 0·06)	0·078	..	..	54	−0·02 (−0·06 to 0·01)	0·154	..	..
Percentage of PWID who are women	83	0·06 (−0·05 to 0·17)	0·276	−0·07 (−0·22 to 0·07)	0·293	63	−0·13 (−0·22 to −0·04)	0·005	−0·14 (−0·26 to −0·02)	0·019
Proportion of the general population aged 15–24 years	90	−0·32 (−0·63 to 0·02)	0·038	−0·06 (−0·44 to 0·31)	0·731	64	0·36 (0·09 to 0·64)	0·011	−0·45 (−0·96 to 0·06)	0·082

n refers to number of countries. For age of PWID and age of onset of IDU, we used a median (range) where available and a mean (range) where a median was not available but a mean was; hence, we refer to these as averages throughout. GDP=gross domestic product. PWID=people who inject drugs.

Higher national youth unemployment was associated with lower age of IDU onset in univariable analyses, but this association was attenuated after adjustment (Table 2, Table 3; figure 3A). No associations were identified between the level of youth unemployment and indicators of youth IDU in univariable and multivariable analyses.

Higher GDP was positively associated with longer duration of IDU and the median age of PWID in both univariable analyses, and multivariable analyses (Table 2, Table 3; figure 3A). No associations were identified between the GDP and other indicators of youth IDU.

Gini coefficient was not associated with any of the indicators of youth IDU in the univariable analyses (Table 2, Table 3; figure 3A).

Higher opioid substitution therapy coverage was positively associated with a longer IDU duration in the univariable analysis, but this association was attenuated in the multivariable model (Table 2, Table 3; figure 3A). No associations were identified between opioid substitution therapy coverage and other indicators of youth IDU. Sensitivity analyses of defining young PWID in different ways (appendix p 49) produced similar results to analyses seen in table 2.

Associations between percentage of young PWID, duration of IDU, age of IDU onset, and average age of IDU with injecting risk and sexual risk behaviours are shown in table 4 and figure 3B. An association was identified between older age of onset of injecting and increased injecting risk behaviours at the country level, but when entered into the multivariable analysis this association was not maintained owing to the strict p value used to account for multiple testing. No further associations were identified between indicators of youth IDU and injecting risk behaviours, and no associations were observed in univariable and multivariable analyses of the relationship to sexual risk behaviour (figure 3B).

**Table 4:** Country-level linear regression analyses of the associations between injecting and sex risk variables and age and injecting history

	**Percentage of PWID with injecting risk**						**Percentage of PWID with sex risk**			
	Univariable analysis (n=70)			Multivariable analysis (n=51)		Univariable analysis (n=59)			Multivariable analysis (n=NA)	
	n	Coefficient (95% CI)	p value	Coefficient (95% CI)	p value	n	Coefficient (95% CI)	p value	Coefficient (95% CI)	p value
Average age	70	0·01 (−0·05 to 0·06)	0·811	..	..	58	−0·00 (−0·07 to 0·06)	0·919	..	..
Duration of injecting	56	−0·03 (−0·09 to 0·02)	0·237	..	..	48	−0·04 (−0·13 to 0·05)	0·410	..	..
Average age of onset of injecting	56	0·06 (0·01 to 0·12)	0·030	0·06 (0·01 to 0·12)	0·030	47	0·04 (−0·05 to 0·13)	0·414	..	..
Percentage of young PWID	55	0·82 (−1·06 to 2·70)	0·384	..	..	46	0·76 (−1·62 to 3·14)	0·523	..	..
Urban population growth	69	0·05 (−0·09 to 0·20)	0·470	..	..	57	−0·02 (−0·20 to 0·16)	0·855	..	..
Youth unemployment	68	−0·00 (−0·02 to 0·02)	0·990	..	..	56	0·01 (−0·02 to 0·03)	0·647	..	..
GDP (per US$1000 increase)	66	0·00 (−0·01 to 0·02)	0·686	..	..	55	0·00 (−0·02 to 0·02)	0·833	..	..
Gini coefficient (per score increase)	67	0·01 (−0·03 to 0·05)	0·594	..	..	55	−0·01 (−0·07 to 0·04)	0·586	..	..
Opioid substitution therapy coverage (per % increase in coverage)	58	0·00 (−0·01 to 0·01)	0·652	..	..	47	−0·00 (−0·01 to 0·01)	0·691	..	..
Percentage of PWID who are women	68	−0·01 (−0·03 to 0·01)	0·341	..	..	56	0·00 (−0·03 to 0·03)	0·960	..	..
Proportion of the general population aged 15–24 years	70	0·00 (−0·07 to 0·06)	0·886	..	..	58	0·02 (−0·06 to 0·11)	0·578	..	..

n refers to number of countries. Injecting risk and sexual risk variables were logit transformed. GDP=gross domestic product. NA=not applicable. PWID=people who inject drugs.

## Discussion

This study identified marked between-country variation in the percentage of PWID aged 25 years or younger; the global estimate is 25·3% (equivalent to approximately 3·9 million people), but between countries these percentages ranged from 7·0% to 50·8%. Half of the estimated PWID population in Latin America are aged 25 years or younger (although only two countries provided data), and around a quarter of the IDU population was aged 25 years or younger in South Asia, eastern Europe, east and southeast Asia, North America, and Australasia. This study is the first to investigate country-level factors underlying this variation, and the first to assess associations between youth IDU and population injecting and sexual risk behaviours. Lower GDP is associated with a shorter median duration of injecting, and with lower median age of the IDU population. Urban population growth is associated with a higher age of onset of IDU and, before adjustment, is associated with a shorter duration of IDU. No associations were observed between indicators of youth IDU and country-level youth unemployment, Gini coefficient, or provision of opioid substitution therapy. Similarly, no indicators of youth IDU were associated with country-level injecting and sexual risk behaviours.

The observed variation in the age profile of PWID is plausibly driven by country-level factors, which warrants consideration of the association between development and IDU. From the results of this study, we hypothesise that countries with lower GDPs are likely to have a lower median age of PWID and shorter duration of IDU, and countries with increased urban population growth are likely to have IDU initiation among older age groups (shorter duration of IDU, but higher age of IDU onset). A projected 68% of the global population will reside in urban areas by 2050, with the majority of this growth expected to be driven by LMICs.^25^ Whether such changes in living and working conditions might unintentionally result in environments that facilitate IDU requires consideration; these changing working and living conditions might represent key factors for prioritisation to optimise the Sustainable Development Goals^26^ for improved mental health.

Several regions with the highest prevalence of PWID aged 25 years or younger also have low coverage of interventions to prevent the spread of blood-borne viruses. In a previous review of global coverage of opioid substitution therapy and needle and syringe programmes, coverage was found to be low in eastern Europe, east and southeast Asia, and North America.^22^ The percentage of young PWID is high in Russia (>50% of the PWID population aged ≤25 years) and the Philippines—countries in which coverage of opioid substitution therapy and needle and syringe programmes is low,^22^ and punitive drug policies create barriers to harm reduction measures.^27^, ^28^ Although our findings indicate that the age profile of PWID is not associated with country-level sexual and injecting risk behaviours (in contrast to previous research of individual-level risk^7^, ^8^) and no evidence indicated that country-level drug policy is driving variation in PWID age, young PWID in countries with low coverage of opioid substitution therapy and needle and syringe programmes will be exposed to greater risk of contracting blood-borne viruses than young PWID in countries with high coverage because of poor access to harm reduction measures. To prevent the epidemic spread of blood-borne viruses among new generations of PWID in countries with a high proportion of young PWID, increasing the public health burden, urgent upscaling of blood-borne viruses prevention coverage is needed in several countries. Outreach interventions might be especially effective for engaging young people with interventions to prevent the spread of blood-borne viruses.^29^

Our study also identified a number of countries with a concerning paucity of data on the age structure of populations of PWID. The countries with data available account for 79% of the global population. However, the highest proportion of countries with no data available were in sub-Saharan Africa (65%), the Caribbean (83%), Latin America (84%), and the Pacific Island States and Territories (100%). Notably, regions with the least available data fit the GDP and urbanisation profile identified in the present analyses as being associated with risk for young PWID, and for recent onset of IDU (shorter duration of IDU and higher age of onset). To monitor these areas, we reiterate calls for improved data transparency associated with IDU and increased epidemiological data investment in areas affected by problematic drug use.^30^

The results of this review and analysis must be considered in the light of several limitations. Heterogeneity was high between studies with regard to the age range used to define young PWID (appendix pp 5–7), with 49 different age range categories identified. To ensure the results reflect the period of adolescence, we excluded any studies that included participants aged 26 and older as their youngest age group from the analyses of the proportion of young PWID, but it is plausible that this heterogeneity contributed to the null results observed for the percentage of young PWID. Excluding studies that included individuals aged 26 and older as the youngest PWID category reduced study power, although sensitivity analyses in which the exclusion age was increased to 30 years and older yielded no substantive differences in results (appendix pp 48–49). All sensitivity analyses are reported in the appendix (pp 47–49).

The original systematic review^1^ included grey literature where available, which should mitigate the effect of publication bias. We have interpreted duration of IDU as a marker of the age of the injecting population (with the rationale that shorter duration will represent a younger population). However, the median duration and population age estimated in this study might be biased, and the PWID within the studies included in the review will not necessarily be representative of those in the overall population, since populations captured in certain study types (eg, opioid substitution therapy centres) might be older than the rest of the population of PWID. Lower median age of PWID populations will plausibly reflect mortality or cessation of IDU rather than bias. Further research assessing the factors that affect the duration of IDU in LMICs is needed to clarify these results.

In our analyses, we were unable to account for heterogeneity at the study level (with the exception of sample size weighting) for injecting duration, median age of PWID, and age at onset of injecting, and were unable to account for heterogeneity at the country level for all indicators of youth IDU. Heterogeneity at the study level was assessed for the percentage of young PWID; only two of 28 studies had an *I*^2^ value of less than 85%. The use of national World Bank indicators in the analysis resulted in the decision to analyse the data at the country level rather than using individual studies as the unit of analysis, which subsequently reduced the study power.

Although our systematic review was completed in 2017,^1^ much of the collected data were older (median publication year 2012). Consequently, the included World Bank and UN figures are only approximations for country-level factors at the time the studies were done. The greatest limitation of our study was that data were missing for many countries; in some regions, no countries contributed data towards regional estimates on youth injecting.

Substantial global variation exists in the age profile of populations of PWID. Young people comprise a large percentage of PWID in several LMICs that have poor coverage of measures to prevent the spread of blood-borne viruses. Such measures are necessary to limit the burden of disease arising from IDU. Without scale-up, the risk of viruses spreading rapidly through new generations in LMICs is high.

Lower GDP and increase in urbanisation are associated with recent uptake of IDU (shorter duration and older age of onset) in the population, with lower GDP countries likely to have younger populations of PWID. Urbanisation might be linked to recent uptake of IDU among older individuals. A better understanding of the origins of these large cross-country differences and the implications for health could be used to improve global development policy.

To increase awareness of emerging trends in drug use among young people, and to respond accordingly to prevent epidemic IDU harms, we reiterate calls for improved data transparency and increased epidemiological monitoring investment in areas affected by problematic drug use.

**This online publication has been corrected. The corrected version first appeared at thelancet.com/lancetgh on Feb 11, 2020**
