# Correction to *Lancet Glob Health* 2020; 8: e76–91

**DOI:** 10.1016/S2214-109X(20)30047-4

**Published:** 2020-02-11

*Hines LA, Trickey A, Leung J, et al. Associations between national development indicators and the age profile of people who inject drugs: results from a global systematic review and meta-analysis.* Lancet Glob Health *2020; **8**: e76–91*—In the Acknowledgments of this Article, the second and third sentences should read “LAH is supported by a Sir Henry Wellcome Postdoctoral Fellowship from the Wellcome Trust. LD is supported by an Australian National Health and Medical Research Council (NHMRC) Senior Principal Research Fellowship.” This correction has been made as of Feb 11, 2020.

