# Supplementary material for: Associations between national development indicators and the age profile of people who inject drugs: results from a global systematic review and meta-analysis
Source: Lancet Glob Health. 2019 Dec 12;8(1):e76–91. doi: 10.1016/S2214-109X(19)30462-0 (PMC7024964; doi:10.1016/S2214-109X(19)30462-0)
Supplement: Supplementary appendix [file mmc1.pdf]

THE LANCET
Global Health

Supplementary appendix

This appendix formed part of the original submission and has been peer reviewed. We post it as supplied by the authors.

Supplement to: Hines LA, Trickey A, Leung J, et al. Associations between national development indicators and the age profile of people who inject drugs: results from a global systematic review and meta-analysis. *Lancet Glob Health* 2020; **8:** e76–91.

Appendices and Supplementary tables

Contents

Page	
2 - 4	Appendix 1: Further details on measures included in analyses
5 - 7	Appendix Table 1: Studies included in the analysis.
8 - 46	Appendix 3: References for studies included in the analysis
47	Supplementary materials: Sensitivity analyses
48	Supplementary table 1: The number and percentage of studies using each youngest age group category
49	Supplementary table 2: Sensitivity analyses for linear regressions of country-level predictors of the % of PWID that are young (logit transformed)

Appendix 1: Further details on measures included in analyses

Measures

Indicators of young PWID

Percentage of PWID that are young

Variable definition

Studies recorded the percentages of PWID (at time of study data collection) in different age categories, for current injectors. To ensure analysis focussed on young people, data from studies including ages above 25 years[21] in their youngest age group were excluded only from analyses of Percentage of PWID that are young. Due to the removal of studies including individuals over age 25 in their "young" age categories, the results for the percentage of PWID that are young differ from those in the paper by Degenhardt et al.[1] Regional and global estimates (for all indicators of young PWID) were weighted by the number of PWID in each country, taken from Degenhardt et al.[1] If a country did not have an estimate for the number of PWID then that country's values for indicators of young PWID would not be counted towards the regional average (mean). The global number of PWID that are young was estimated by multiplying the global estimate of 15·6 million PWID by the estimate for the percentage of PWID that are young (and the 95% Uncertainty Intervals – a Monte Carlo simulated interval taking 100,000 draws).

Available data

In total, 49 different age categories were used between studies to define PWID as young. Data from studies including ages above 25 years (n=105) in their youngest age group were excluded from analyses of this variable. After this exclusion, data on the percentage of PWID that are young were available for 259 studies. From 60 countries, and the most common age category for the youngest age group was age 18-24, used in 24% of the studies (see supplementary table 1). The lowest maximum age for the young PWID age group in a study was 17, whilst the highest was 25.

Median duration of IDU

Variable definition

Where possible, duration of IDU (at time of study data collection) was taken from figures reported in studies. Where not directly reported, duration of IDU was calculated by subtracting average reported age of IDU onset from average age at study participation (see below) for studies not reporting duration.

Available data

Median duration of injecting data were available for 380 studies, from 68 countries.

Average age of PWID

Variable definition

Average age of PWID (at time of study data collection) was used where reported. A median was used where available, otherwise a mean value was used, so we refer to this value as an average. In studies where these data were not reported, a median age was imputed by using the age of onset and the duration of injecting.

Available data

Of the 741 studies, 595 reported a mean or median age of PWID, whilst a median age was imputed for the other 146 studies by using the age of onset and the duration of injecting.

Age of IDU onset

Variable definition

The average age of IDU onset (at time of study data collection) was used if directly reported. A median was used where available and a mean if not. If neither was directly reported, average age of onset of injecting was imputed subtracting average duration of injection from participants' average age at the time of data collection.

Available data

The average age of onset of injecting (at time of study data collection) was reported in 384 studies encompassing 66 countries. Of these studies, 212 had this information directly reported. Average age of onset of injecting was imputed for the other 172 by subtracting average duration of injection from participants' average age at the time of data collection.

Country-level sociodemographic and economic factors

To examine whether measures of youth IDU were associated with sociodemographic and wealth indicators, the following measures were derived from World Bank Figures for 2015:[15] GDP per capita (in thousand $USD), youth unemployment (proportion of labour force aged 15-24), urban population growth (annual percentage change, measuring the change in the percentage of the country's population living in urban areas), GINI coefficient (measuring inequality on a scale from 0-100, with lower as more equal), and the proportion of the general population aged 15-24.[16] Data from prior meta-analyses were used for

measures of Opioid Substitution Treatment (OST) coverage[22] (per PWID per year) and the percentage of female PWID in a country.[23]

High-risk injecting and sex behaviours

Variable definition

We also report pooled estimate of the percentage of PWID who had recently engaged in injecting risk behaviour (predominantly receptive needle sharing, typically in the past month) and pooled estimated of the percentage of PWID who recently engaged in sexual risk behaviour (predominantly no or inconsistent condom use with casual partner, typically within the past month).

Available data

Information on high-risk injecting and sex behaviour was available for 70 and 59 countries, respectively.

Appendix Table 1: Studies included in the analysis.

Country	Study publication year(s)	References	I^2 value for the percentage of PWID that are young
Afghanistan	**2008, 2010-2012, 2014, 2015**	**[1-8]**	**60.0%**
Algeria	**2014**	**[9]**	**NA**
Armenia	**2011, 2013**	**[10,11]**	**NA**
Australia	**2008-2016**	**[12-37]**	**99.0%**
Austria	**2012, 2014**	**[38,39]**	**NA**
Azerbaijan	**2012**	**[40]**	**NA**
Bangladesh	**2008, 2011, 2015, 2016**	**[41-45]**	**NA**
Belarus	**2015**	**[46]**	**96.7%**
Belgium	**2010-2015**	**[47-52]**	**NA**
Benin	**2014, 2015**	**[53,54]**	**NA**
Bosnia and Herzegovina	**2012**	**[55]**	**94.5%**
Bulgaria	**2012, 2014, 2015**	**[56-58]**	**NA**
Cambodia	**2010, 2012**	**[59,60]**	**NA**
Canada	**2008-2016**	**[61-80]**	**99.4%**
China	**2008-2017**	**[81-138]**	**97.6%**
China, Hong Kong SAR	**2008**	**[139]**	**NA**
Colombia	**2011, 2016**	**[140-142]**	**90.5%**
Croatia	**2009-2011, 2016**	**[143-147]**	**NA**
Cyprus	**2010, 2012**	**[148,149]**	**NA**
Czech Republic	**2011, 2017**	**[150,151]**	**NA**
Côte d'Ivoire	**2016**	**[152]**	**NA**
Democratic Republic of the	**2015**	**[153]**	**NA**
Denmark	**2011, 2014**	**[154-156]**	**NA**
England	**2009, 2011, 2012, 2014**	**[157-162]**	**NA**
Estonia	**2008, 2010, 2012-2014, 2016**	**[163-172]**	**94.8%**
Georgia	**2009-2012, 2014, 2015**	**[173-178]**	**89.7%**
Germany	**2009, 2011, 2014, 2016**	**[179-185]**	**89.9%**
Ghana	**2016**	**[186]**	**NA**
Greece	**2010, 2012, 2013, 2015**	**[187-193]**	**NA**
Hungary	**2009-2013, 2015, 2017**	**[194-203]**	**96.5%**
Iceland	**2015**	**[204]**	**NA**
India	**2008-2017**	**[205-236]**	**99.4%**
Indonesia	**2009-2013, 2015**	**[237-244]**	**NA**
Iran (Islamic Republic of)	**2008-2010, 2012-2017**	**[245-268]**	**94.6%**
Iraq	**2015**	**[252]**	**NA**
Ireland	**2010, 2013**	**[269,270]**	**NA**
Israel	**2010**	**[271]**	**NA**
Italy	**2008, 2010, 2011, 2014**	**[272-275]**	**NA**

Kazakhstan	**2010**	[276]	**NA**
Kenya	**2011, 2012, 2015**	[277-283]	**63.7%**
Korea (Republic of)	**2008, 2013**	[284,285]	**NA**
Kyrgyzstan	**2010, 2013, 2016**	[276,286,287]	**NA**
Lao People's Democratic	**2011**	[288]	**NA**
Latvia	**2009, 2013, 2014**	[166,289,290]	**NA**
Lebanon	**2010, 2016**	[291,292]	**NA**
Libyan Arab Jamahiriya	**2013**	[293]	**NA**
Lithuania	**2009, 2014**	[290,294]	**NA**
Macedonia (TFYR)	**2013, 2014**	[295,296]	**NA**
Madagascar	**2012, 2014**	[297,298]	**99.5%**
Malaysia	**2009, 2012, 2015**	[299-301]	**NA**
Maldives	**2008**	[302]	**NA**
Mauritius	**2011**	[303,304]	**NA**
Mexico	**2008, 2009, 2016**	[305-308]	**NA**
Moldova (Republic of)	**2013**	[309]	**93.4%**
Montenegro	**2009, 2013**	[310,311]	**NA**
Morocco	**2014**	[312,313]	**NA**
Mozambique	**2015**	[314]	**NA**
Myanmar	**2010-2015**	[109,315-322]	**93.4%**
Nepal	**2009-2016**	[323-338]	**98.7%**
New Zealand	**2010, 2014-2017**	[339-343]	**NA**
Nicaragua	**2014**	[344]	**NA**
Nigeria	**2010, 2013, 2014**	[345-347]	**NA**
Norway	**2009, 2010, 2014, 2015**	[348-351]	**NA**
Occupied Palestinian	**2010, 2016**	[352,353]	**NA**
Pakistan	**2008, 2009, 2012, 2013, 2015,**	[354-361]	**96.1%**
Philippines	**2009, 2011, 2013**	[362-364]	**99.5%**
Poland	**2011, 2013, 2014, 2015**	[365-369]	**NA**
Portugal	**2011**	[370]	**NA**
Puerto Rico	**2010, 2015, 2016**	[205,371-373]	**NA**
Romania	**2011, 2012, 2013, 2014, 2015**	[374-379]	**NA**
Russian Federation	**2008, 2009, 2011, 2013**	[380-389]	**NA**
Saudi Arabia	**2015**	[390]	**NA**
Scotland	**2008, 2010-2012, 2014, 2015**	[391-397]	**95.5%**
Serbia	**2009, 2014**	[311,398,399]	**97.8%**
Seychelles	**2011**	[400]	**NA**
Sierra Leone	**2013**	[401]	**NA**
Singapore	**2015**	[402]	**NA**
Slovakia	**2012**	[403]	**NA**
Slovenia	**2013**	[404]	**NA**
Spain	**2008, 2010-2014, 2017**	[405-417]	**99.9%**
Sri Lanka	**2015**	[418]	**NA**
Sweden	**2011, 2012, 2014, 2015, 2017**	[419-425]	**NA**
Switzerland	**2016**	[426]	**NA**

Syrian Arab Republic	**2014**	[427]	**NA**
Taiwan, Province of China	**2011-2014**	[428-433]	**NA**
Tajikistan	**2009, 2010, 2016**	[276,434,435]	**NA**
Tanzania (United Republic	**2008, 2014-2016**	[436-443]	**98.2%**
Thailand	**2008, 2010-2012, 2015, 2016**	[444-451]	**98.4%**
Togo	**2015**	[452]	**NA**
Tunisia	**2009, 2015**	[453,454]	**NA**
Turkey	**2012, 2016**	[455,456]	**NA**
Ukraine	**2008, 2009, 2010, 2012, 2014,**	[457-466]	**99.3%**
United States of America	**2008-2017**	[305,372,467-505]	**98.4%**
Viet Nam	**2008, 2010, 2011, 2014-2016**	[506-514]	**98.3%**
Wales	**2010**	[515]	**NA**

Appendix 3: References for studies included in the analysis

1. World Bank. Mapping and Situation Assessment of Key Populations at High Risk of HIV in Three Cities of Afghanistan. 2008.
2. National AIDS Control Program Afghanistan. Integrated Biological & Behavioral Surveillance (IBBS) in Selected Cities of Afghanistan. 2012.
3. Todd CS, Nasir A, Stanekzai M, et al. Prevalence and correlates of HIV, syphilis, and hepatitis B and C infection and harm reduction program use among male injecting drug users in Kabul, Afghanistan: A cross-sectional assessment. *Harm Reduction Journal* 2011; **8**: 22.
4. Todd CS, Nasir A, Raza Stanekzai M, et al. Prevalence and correlates of syphilis and condom use among male injection drug users in four Afghan cities. *Sexually Transmitted Diseases* 2010; **37**(11): 719-25.
5. Ruisenor-Escudero H, Vu A, Wirtz AL, et al. Cross-sectional assessments of participants' characteristics and loss to follow-up in the first Opioid Substitution Therapy Pilot Program in Kabul, Afghanistan. *Harm Reduction Journal* 2015; **12 (1) (no pagination)**(28).
6. Bautista CT, Todd CS, Abed AMS, et al. Effects of duration of injection drug use and age at first injection on HCV among IDU in Kabul, Afghanistan. *Journal of Public Health* 2010; **32**(3): 336-41.
7. Nasir A, Todd CS, Stanekzai MR, et al. Implications of hepatitis C viremia vs. antibody alone on transmission among male injecting drug users in three Afghan cities. *International Journal of Infectious Diseases* 2011; **15**(3): e201-5.
8. Ruisenor-Escudero H. Injecting drug use in Afghanistan: Risk factors for HIV, HCV, STIS, injecting drug practices, and outcomes of an opiate substitution therapy program. *Dissertation Abstracts International: Section B: The Sciences and Engineering* 2014; **75**(2-B(E)): No Pagination Specified.
9. ONUSIDA. Rapport d'Activite sur la Riposte Nationale au VIH/Sida. Report to UNAIDS. 2014.
10. Grigoryan S, Hakobyan A, Papoyan A, et al. Results from the HIV Biological and Behavioural Surveillance in the Republic of Armenia. 2013.
11. Global Fund. Armenia IBBS. 2012.
12. Cama E, Brener L, Wilson H, von Hippel C. Internalized stigma among people who inject drugs. *Substance Use & Misuse* 2016; **51**(12): 1664-8.
13. KPMG. Further evaluation of the Medically Supervised Injecting Centre during its extended Trial period (2007-2011). 2010.
14. Stafford J, Breen C. Australian Drug Trends 2015: Findings from the Illicit Drug Reporting System (IDRS). *Australian Drug Trends Series* 2016; **145**.

15. Larance B, Lintzeris N, Ali R, et al. The diversion and injection of a buprenorphine-naloxone soluble film formulation. *Drug & Alcohol Dependence* 2014; **136**: 21-7.

16. Fisher K, Smith T, Nairn K, Anderson D. Rural people who inject drugs: A cross-sectional survey addressing the dimensions of access to secondary needle and syringe program outlets. *the Australian Journal of Rural Health* 2016.

17. The Kirby Institute. Prevalence of HIV, HCV and injecting and sexual behaviour among Needle and Syringe Program attendees 1995-2014. 2015.

18. Australian Government Department of Health. National Drug Strategy Household Survey. 2013.

19. O'Brien S, Day C, Black E, Dolan K. Injecting drug users' understanding of hepatitis C. *Addictive Behaviors* 2008; **33**(12): 1602-5.

20. McKetin R, Ross J, Kelly E, et al. Characteristics and harms associated with injecting versus smoking methamphetamine among methamphetamine treatment entrants. *Drug & Alcohol Review* 2008; **27**(3): 277-85.

21. Winstock AR, Lea T, Sheridan J. Prevalence of diversion and injection of methadone and buprenorphine among clients receiving opioid treatment at community pharmacies in New South Wales, Australia. *International Journal of Drug Policy* 2008; **19**(6): 450-8.

22. Wilson H, Brener L, Mao L, Treloar C. Perceived discrimination and injecting risk among people who inject drugs attending Needle and Syringe Programmes in Sydney, Australia. *Drug and Alcohol Dependence* 2014; **144**: 274-8.

23. Salmon AM, Van Beek I, Amin J, Grulich A, Maher L. High HIV testing and low HIV prevalence among injecting drug users attending the Sydney Medically Supervised Injecting Centre. *Australian and New Zealand Journal of Public Health* 2009; **33**(3): 280-3.

24. Paquette DM, Bryant J, Crawford S, de Wit JB. Conducting a respondent-driven sampling survey with the use of existing resources in Sydney, Australia. *Drug and Alcohol Dependence* 2011; **116**(1-3): 125-31.

25. Laslett AM, Dietze P, Dwyer R. The oral health of street-recruited injecting drug users: Prevalence and correlates of problems. *Addiction* 2008; **103**(11): 1821-5.

26. Kerr D, Dietze P, Kelly AM, Jolley D. Attitudes of Australian heroin users to peer distribution of naloxone for heroin overdose: Perspectives on intranasal administration. *Journal of Urban Health* 2008; **85**(3): 352-60.

27. Islam M, Stern T, Conigrave KM, Wodak A. Client satisfaction and risk behaviours of the users of syringe dispensing machines: a pilot study. *Drug & Alcohol Review* 2008; **27**(1): 13-9.

28. Gibbie TM, Hides LM, Cotton SM, Lubman DI, Aitken C, Hellard M. The relationship between personality disorders and mental health, substance use severity and quality of life among injecting drug users. *Medical Journal of Australia* 2011; **195**(3 SUPPL.): S16-S21.
29. Fisher DG, Wilson H, Bryant J. Harm reduction knowledge and information exchange among secondary distributors in Sydney, Australia. *Drugs: Education, Prevention & Policy* 2013; **20**(1): 67-73.
30. Dwyer R, Topp L, Maher L, et al. Prevalences and correlates of non-viral injecting-related injuries and diseases in a convenience sample of Australian injecting drug users. *Drug and Alcohol Dependence* 2009; **100**(1-2): 9-16.
31. Day C, White B, Thein H, et al. Experience of hepatitis C testing among injecting drug users in Sydney, Australia. *AIDS Care* 2008; **20**(1): 116-23.
32. Darke S, Torok M. The association of childhood physical abuse with the onset and extent of drug use among regular injecting drug users. *Addiction* 2014; **109**(4): 610-6.
33. Conroy E, Kimber J, Dolan K, Day C. An examination of the quality of life among rural and outer metropolitan injecting drug users in NSW, Australia. *Addiction Research & Theory* 2008; **16**(6): 607-17.
34. Bryant J, Paquette D, Wilson H. Syringe coverage in an Australian setting: does a high level of syringe coverage moderate syringe sharing behaviour? *AIDS & Behavior* 2012; **16**(5): 1156-63.
35. Sacks-Davis R, Daraganova G, Aitken C, et al. Hepatitis C virus phylogenetic clustering is associated with the social-injecting network in a cohort of people who inject drugs. *PLoS ONE [Electronic Resource]* 2012; **7**(10): e47335.
36. Deacon RM, Topp L, Wand H, et al. Correlates of susceptibility to hepatitis B among people who inject drugs in Sydney, Australia. *Journal of Urban Health* 2012; **89**(5): 769-78.
37. Miller ER, Hellard ME, Bowden S, Bharadwaj M, Aitken CK. Markers and risk factors for HCV, HBV and HIV in a network of injecting drug users in Melbourne, Australia. *Journal of Infection* 2009; **58**(5): 375-82.
38. Reitox National Focal Point, Weigl M, Busch M, Grabenhofer-Eggerth A, Turscherl E, Wirl C. 2011 National Report (2010 data) to the EMCDDA. Austria: New developments, trends and in-depth information on selected issues, 2012.
39. Reitox National Focal Point, Weigl M, Busch M, et al. 2013 National Report (2012 data) to the EMCDDA. Austria: New Development and Trends, 2014.
40. WHO. The report on results of a surveillance survey on knowledge, risks and prevalence of HIV and sexually and parenterally transmitted infections in most-at-risk populations in Azerbaijan. 2012.
41. National AIDS/STD Program Bangladesh. Behavioral Surveillance Survey 2006-07: Technical Report. 2008.

42. National AIDS/STD Programme Bangladesh. Mapping Study and Size Estimation of Key Populations in Bangladesh. 2016.
43. Azim T, Rahman M, Rahman M, et al. National HIV Serological Surveillance, 2011 Bangladesh. 2011.
44. Hossain KJ, Nandi AK. Hepatitis-B Infections among the Injection Drug Abusers: An Emerging Risk in Public Health, Bangladesh. *Mymensingh Medical Journal: MMJ* 2015; **24**(4): 813-24.
45. Azim T, Chowdhury EI, Reza M, et al. Prevalence of infections, HIV risk behaviors and factors associated with HIV infection among male injecting drug users attending a needle/syringe exchange program in Dhaka, Bangladesh. *Substance Use & Misuse* 2008; **43**(14): 2124-44.
46. Arkad'yevna YK. ПОВЕДЕНЧЕСКИЕ ОСОБЕННОСТИ И УРОВЕНЬ ЗНАНИЙ ПО ПРОБЛЕМЕ ВИЧ/СПИД СРЕДИ ПОТРЕБИТЕЛЕЙ ИНЪЕКЦИОННЫХ НАРКОТИКОВ. 2015.
47. Reitox National Focal Point, Deprez N, Antoine J, et al. 2011 National Report (2010 data) to the EMCDDA. Belgium: New developments, trends and in-depth information on selected issues. 2012.
48. Reitox National Focal Point, Plettinckx E, Antoine J, Bollaerts K, Blanckaert P, Van Bussel JCH. 2012 National Report (2011 data) to the EMCDDA. "Belgium" New Development, Trends and in-depth information on selected issues, 2013.
49. Reitox National Focal Point, Lamkaddem B, Roelands M. 2010 National Report (2009 data) to the EMCDDA. Belgium: New developments, trends and in-depth information on selected issues: Scientific Institute of Public Health, 2011.
50. Reitox National Focal Point, Plettinckx E, Antoine J, Blanckaert P, van Bussel JCH. 2013 National Report (2012 data) to the EMCDDA "Belgium" New Development, Trends and in-depth information on selected issues: Belgian Monitoring Centre for Drugs and Drug Addiction

Scientific Institute of Public Health OD Public health and Surveillance, 2014.
51. Reitox National Focal Point, Antoine J, Blanckaert P, et al. Belgian National Report on drugs 2014, 2015.
52. Lamkaddem B, Roelands M, Deprez N. Belgium: New developments, trends and in-depth information on selected issues. 2009 National Report to the EMCDDA, 2010.
53. Plan Benin. Benin Consommateurs de Drogues Injectables (CDI). 2015.
54. Plan Bénin. Enquête de surveillance de deuxième génération du VIH et des IST auprès des utilisateurs de drogues injectables au Bénin. 2014.
55. Bacak V, Dominkovic Z. Report on behavioral and biological surveillance among injection drug users in Bosnia and Herzegovina, 2009: a respondent driven sampling survey, 2009. 2012.

56. Reitox National Focal Point. 2011 National Report (2010 data) to the EMCDDA. Bulgaria: New Development, Trends and in-depth information on selected issues, 2012.
57. Reitox National Focal Point. 2013 National Report (2012 data) to the EMCDDA. Bulgaria: New Development, Trends and in-depth information on selected issues, 2014.
58. Reitox National Focal Point. 2014 National Report (2013 data) to the EMCDDA. Bulgaria: New Development, Trends and in-depth information on selected issues, 2015.
59. Chhea C, Seguy N. HIV prevalence among drug users in Cambodia 2007. *NCHADS & NACD* 2010.
60. Chhorvann C, Sopheab H, Sovannary T. National Population Size Estimation, HIV Related Risk Behaviors and HIV Prevalence among People Who Use Drugs in Cambodia. 2012.
61. Hennink M, Abbas Z, Lloyd K, Population and Public Health Services. Injecting and Sexual Risk Behviors Among Persons Who Inject Drugs in the Regina Qu'Appelle Health Region: Results from the I-Track Surveys, 2011.
62. Gratrix J, Plitt S, Singh AE, Edmonton Site I-Track Phase 3 Investigators. I-Track Phase 3: Enhanced Surveillance of Risk Behaviours among People who Inject Drugs in Canada (Edmonton Site). 2014.
63. Millson P, White S, Leonard L, Public Health Agency of Canada. Enhanced Surveillance of Risk Behaviours and Prevalence of HIV and Hepatitis C among People who Inject Drugs, 2016.
64. Machalek K, Hanley BE, Bacon P. Whitehorse I-Track Report: Monitoring Behaviour among People Who Inject or Inhale Drugs in Whitehorse, Yukon. Blood Ties Four Directions Centre, 2014.
65. Shoemaker M, Taylor L, Callaghan R. Prince George 2012 I-Track Survey Results: Findings and Discussion, 2013.
66. Caldarelli H, Locker A, Warshawksy B. I-Track Survey, Phase 3: A Profile of People Who Inject Drugs in London, Ontario, 2013.
67. Leclerc P, Roy E, Morissette C, Alary M, Parent R, Blouin K. Surveillance des maladies infectieuses chez les utilisateurs de drogue par injection: Epidemiologie du VIH/VHC de 1995 a 2014. 2016.
68. Public Health Agency of Canada. I-Track: Enhanced Surveillance of HIV, Hepatitis C and associated risk behaviours among people who inject drugs in Canada. Phase 2 Report. 2014.
69. Lloyd-Smith E, Wood E, Zhang R, Tyndall MW, Montaner JS, Kerr T. Determinants of Cutaneous Injection-Related Infection Care at a Supervised Injecting Facility. *Annals of Epidemiology* 2009; **19**(6): 404-9.

70. Strike C, Kolla G, Balian R, et al. HIV prevention intervention targets - Injection Initiation and Modeling Behaviour. *Canadian Journal of Infectious Diseases and Medical Microbiology* 2010; **SB)**: 88B.

71. Shaw SY, Shah L, Jolly AM, Wylie JL. Identifying heterogeneity among injection drug users: A cluster analysis approach. *American Journal of Public Health* 2008; **98**(8): 1430-7.

72. Shaw A, Lazarus L, Pantalone T, et al. Risk environments facing potential users of a supervised injection site in Ottawa, Canada. *Harm Reduction Journal* 2015; **12**: 49.

73. Ivsins A, Chow C, Macdonald S, et al. An examination of injection drug use trends in Victoria and Vancouver, BC after the closure of Victoria's only fixed-site needle and syringe programme. *International Journal of Drug Policy* 2012; **23**(4): 338-40.

74. Gibson EK, Exner H, Stone R, Lindquist J, Cowen L, Roth EA. A mixed methods approach to delineating and understanding injection practices among clientele of a Victoria, British Columbia needle exchange program. *Drug and Alcohol Review* 2011; **30**(4): 360-5.

75. Cox J, De P, Morissette C, et al. Low perceived benefits and self-efficacy are associated with hepatitis C virus (HCV) infection-related risk among injection drug users. *Social Science and Medicine* 2008; **66**(2): 211-20.

76. Bruneau J, Daniel M, Kestens Y, Abrahamowicz M, Zang G. Availability of body art facilities and body art piercing do not predict hepatitis C acquisition among injection drug users in Montreal, Canada: Results from a cohort study. *International Journal of Drug Policy* 2010; **21**(6): 477-84.

77. Bertard MC, Gagne C, Mercure SA, Gagnon M, Godin G, Cote F. [Psychosocial determinants of drug users to use a new syringe]. *Revue d Epidemiologie et de Sante Publique* 2010; **58**(3): 197-205.

78. Shaw SY, Jolly AM, Wylie JL. Outlier populations: individual and social network correlates of solvent-using injection drug users. *PLoS ONE [Electronic Resource]* 2014; **9**(2): e88623.

79. Rashidi B, Tossonian H, Sharma S, et al. Engaging high risk populations of downtown vancouver through Hepatitis C and HIV portable pop-up clinics. *Canadian Journal of Infectious Diseases and Medical Microbiology* 2014; **25**: 19A.

80. Leclerc P, Roy E, Morissette C, Vaillancourt E. HIV and HCV prevalence among cocaine users in Montreal. *Canadian Journal of Infectious Diseases and Medical Microbiology* 2012; **23**: 82A.

81. Chen F, Zhang J, Guo F, et al. Hepatitis B, C, and D virus infection showing distinct patterns between injection drug users and the general population. *Journal of Gastroenterology and Hepatology (Australia)* 2017; **32**(2): 515-20.

82. Zhou Y, Luo W, Cao XB, Zhang B, Wu ZY. [Overdose of heroin and influencing factors in intravenous drug users in parts of Yunnan]. *Chung-Hua Liu Hsing Ping Hsueh Tsa Chih Chinese Journal of Epidemiology* 2016; **37**(5): 648-52.

83. Hao QX. Survey on Infection of HIV and HCV Among 277 Cases of Methadone Maintenance Treatment. 2009.
84. Su MF. HIV, HCV and TB infection among drug users receiving methadone treatment in Yuhuan country, Zhejiang province. *Disease Surveillance* 2010.
85. Wang DY, Chen YM, Huang QL, Zhou M, Chen HM, Pan L. Infection and Risk Factors of HIV and HCV among Drug Users in Zigong City. 2009.
86. Wang J, Yang XJ. Prevalence of HIV, HCV Infection and Syphilis among Drug Users at Methadone Maintenance Clinics in Anshun. 2010.
87. Xia L, Zhang QS, Deng XL, et al. Prevalence of HIV, HCV and Syphilis among Heroin Addicts at Methadone Maintenance Treatment Clinic in Shenzhen City. 2010.
88. Yao W, Jiang ZH, Jia SG, Yang H, Liao KK. Prevalence of HIV, HCV and Syphilis Infection at Methadone Maintenance Treatment Clinic in Mianyang City. 2008.
89. Zhang Q, Wu T, Huang Y, Tan DY, Xi DR. The Analysis of Sentinel Surveillance among Drug Users from 2006 to 2010 in Shunqing District of Nanchong City. 2011.
90. Zhu XH, Xun JP, Gao L, Peng JJ. Monitoring and Analysis of Clinical Blood in 613 MMT Clients. 2010.
91. Deng X. Analysis of HIV and HPC in drug users (in Mandarin). 2014.
92. Zhou Y. 2009—2011 年佛山市顺德区吸毒人群艾滋病 监测结果分析. 2013.
93. Li N, Wang XW, Nie YG, et al. HCV infection status and related risk factors in drug users under HIV sentinel surveillance in Henan province, 2011-2015. *Zhonghua liu xing bing xue za zhi = Zhonghua liuxingbingxue zazhi* 2016; **37**(6): 821-5.
94. Du W, Xiang Y, Wang Z, et al. Socio-demographic and clinical characteristics of 3129 heroin users in the first methadone maintenance treatment clinic in China. *Drug and Alcohol Dependence* 2008; **94**: 158-64.
95. Bai Y, Lai WS, Wei L. Prevalence of HIV, HCV and Syphilis Infection at Methadone Maintenance Treatment Clinic in Liuzhou City. 2009.
96. G. D, H. J. HIV, HCV and Syphilis Infection among Drug Users in Luzhou City. 2009.
97. Gao LF, Yang J, Li SJ. Detection and Analysis of HIV, HBV, Syphilis and HCV Infections of Methadone Clinic in Jinchang City. 2010.
98. Han XJ, Xu YX, Jiang HM, He J, Tan B, Cao FB. Infection of HIV,HBV,HCV and Syphilis in 300 Drug Abusers in Guiyang City. 2010.
99. Lan GH. The Analysis on HIV Surveillance among Guangxi IDUs in 2005-2007. 2008.
100. Chen LF. Survey of Infectious Status of HIV, HBV, Syphilis and HCV on 459 Drug Users. 2009.

101. Wang, Chen. 年四川省达州市吸毒人群艾滋病哨点监测结果分析. 2014.

102. Wang Y, Guan Y. 乌鲁木齐市静脉吸毒人群艾滋病病毒 感染及危险因素研究. 2009.

103. Wen J. 2009 年桂林市静脉注射吸毒人员基线调查报告. 2009.

104. Xu, Chu. 昆明市 E _年吸毒人员 TV 感染及相关因素分析. 2011.

105. Zhang, Gong. 第四轮中国全球基金/中英艾滋病项目 部分项目县(吸毒人群艾滋病行为学监测结果分析. 2009.

106. Zhao G. 1996-2009 年云南省玉溪市艾滋病 吸毒哨点监测结果分析, 2010.

107. Tan, Zhou. 南宁市 2007 —2008 年静脉吸毒人群艾滋病性病血清检测结果分析. 2010.

108. Zhou JS, Zhang KL, Zhang LL, et al. A quasi-experimental study on a community-based behaviour change programme among injecting drug users in Sichuan, China. *International Journal of STD and AIDS* 2009; **20**(2): 125-9.

109. Zhou YH, Liu FL, Yao ZH, et al. Comparison of HIV-, HBV-, HCV- and Co-infection prevalence between Chinese and Burmese intravenous drug users of the China-Myanmar border region. *PloS one* 2011; **6 (1) (no pagination)**(e16349).

110. Zhou YH, Yao ZH, Liu FL, et al. High prevalence of HIV, HCV, HBV and co-infection and associated risk factors among injecting drug users in Yunnan Province, China. *PloS one* 2012; **7 (8) (no pagination)**(e42937).

111. Zhang L, Li J, Lai W, et al. Prevalence and correlates of needle-sharing among new and long-term injection drug users in southwest China. *Substance Use & Misuse* 2010; **45**(14): 2503-23.

112. Yin L, Zhang Y, Qian HZ, et al. Willingness of Chinese injection drug users to participate in HIV vaccine trials. *Vaccine* 2008; **26**(6): 762-8.

113. Yang Y, Latkin C, Luan R, Yang C. Reality and feasibility for pharmacy-delivered services for people who inject drugs in Xichang, China: Comparisons between pharmacy staff and people who inject drugs. *International Journal of Drug Policy* 2016; **27**: 113-20.

114. Williams CT, Liu W, Levy JA. Crossing over: drug network characteristics and injection risk along the China-Myanmar border. *AIDS & Behavior* 2011; **15**(5): 1011-6.

115. Wang Z, Du J, Sun H, Wu H, Xiao Z, Zhao M. Patterns of childhood trauma and psychological distress among injecting heroin users in China. *PloS one* 2010; **5**(12).

116. Wang M, Mao W, Zhang L, et al. Methadone maintenance therapy and HIV counseling and testing are associated with lower frequency of risky behaviors among injection drug users in China. *Substance Use & Misuse* 2015; **50**(1): 15-23.
117. Wang K, Fu H, Longfield K, Modi S, Mundy G, Firestone R. Do community-based strategies reduce HIV risk among people who inject drugs in China? A quasi-experimental study in Yunnan and Guangxi provinces. *Harm Reduction Journal* 2014; **11**: 15.
118. Li J, Gu J, Lau JT, Chen H, Mo PK, Tang M. Prevalence of depressive symptoms and associated factors among people who inject drugs in China. *Drug and Alcohol Dependence* 2015; **151**: 228-35.
119. Lau JTF, Zhang L, Zhang Y, et al. Changes in the prevalence of HIV-related behaviors and perceptions among 1832 injecting drug users in Sichuan, China. *Sexually Transmitted Diseases* 2008; **35**(4): 325-35.
120. Lau JT, Tsui HY, Zhang Y, et al. Comparing HIV-related syringe-sharing behaviors among female IDU engaging versus not engaging in commercial sex. *Drug and Alcohol Dependence* 2008; **97**(1-2): 54-63.
121. Gu J, Wang R, Chen H, et al. Prevalence of needle sharing, commercial sex behaviors and associated factors in Chinese male and female injecting drug user populations. *AIDS Care* 2009; **21**(1): 31-41.
122. Du J, Lombardi C, Evans E, Jiang H, Zhao M, Meng YY. A mixed methods approach to identifying factors related to voluntary HIV testing among injection drug users in Shanghai, China. *International Journal of Infectious Diseases* 2012; **16**(7): e498-e503.
123. Chen HT, Tuner N, Chen CJ, Lin HY, Liang S, Wang S. Correlations between compulsory drug abstinence treatments and HIV risk behaviors among injection drug users in a border city of South China. *AIDS Education and Prevention* 2013; **25**(4): 336-48.
124. Wu Q, Zu J, Wei X, et al. Survey of Hepatitis B infection and vaccination status among drug users in Xi'an. *Chung-Hua Yu Fang i Hsueh Tsa Chih [Chinese Journal of Preventive Medicine]* 2014; **48**(10): 862-6.
125. Wong NS, Chan PC, Lee SS, Lee SL, Lee CK. A multilevel approach for assessing the variability of hepatitis C prevalence in injection drug users by their gathering places. *International Journal of Infectious Diseases* 2013; **17**(3): e193-8.
126. Tao YL, Tang YF, Qiu JP, et al. Prevalence of hepatitis C infection among intravenous drug users in Shanghai. *World Journal of Gastroenterology* 2013; **19**(32): 5320-5.
127. Hser Y, Du J, Li J, et al. Hepatitis C among methadone maintenance treatment patients in Shanghai and Kunming, China. *Journal of Public Health* 2012; **34**(1): 24-31.

128. Gupta S, Iudicello JE, Shi C, et al. Absence of neurocognitive impairment in a large Chinese sample of HCV-infected injection drug users receiving methadone treatment. *Drug & Alcohol Dependence* 2014; **137**: 29-35.
129. Gong JM, Li L, Wang HP, Tang WM, Yang HT, Zhang Y. Correlated factors of hepatitis C virus infection among injection drug users. *Pharmaceutical Biotechnology* 2011; **18**(1): 61-5.
130. Cao H, Zhang K, Shu X, Xu Q, Li G. Detection of hepatitis C core antigen in intravenous drug addictions. *Chinese Journal of Experimental & Clinical Virology* 2011; **25**(4): 304-6.
131. Bao Y, Du C, Lu H, et al. The investigation of HIV and HCV infection and risk factors among opiate drug users in Beijing, China. *American Journal of Drug & Alcohol Abuse* 2012; **38**(2): 140-5.
132. Zhang L, Zhang D, Chen W, Zou X, Ling L. High prevalence of HIV, HCV and tuberculosis and associated risk behaviours among new entrants of methadone maintenance treatment clinics in Guangdong Province, China. *PLoS ONE [Electronic Resource]* 2013; **8**(10): e76931.
133. Yao Y, Wang N, Chu J, et al. Sexual behavior and risks for HIV infection and transmission among male injecting drug users in Yunnan, China. *International Journal of Infectious Diseases* 2009; **13**(2): 154-61.
134. Wu J, Huang J, Xu D, Lu C, Deng X, Zhou X. Infection status and risk factors of HIV, HBV, HCV, and syphilis among drug users in Guangdong, China--a cross-sectional study. *BMC Public Health* 2010; **10**: 657.
135. Luo W, Wu Z, Poundstone K, et al. Needle and syringe exchange programmes and prevalence of HIV infection among intravenous drug users in China. *Addiction* 2015; **110**(Suppl 1): 61-7.
136. Li L, Assanangkornchai S, Duo L, McNeil E, Li J. Risk behaviors, prevalence of HIV and hepatitis C virus infection and population size of current injection drug users in a China-Myanmar border city: results from a Respondent-Driven Sampling Survey in 2012. *PLoS ONE [Electronic Resource]* 2014; **9**(9): e106899.
137. Kretzschmar M, Zhang W, Mikolajczyk RT, et al. Regional differences in HIV prevalence among drug users in China: potential for future spread of HIV? *BMC Infectious Diseases* 2008; **8**: 108.
138. Jia Y, Lu F, Zeng G, et al. Predictors of HIV infection and prevalence for syphilis infection among injection drug users in China: Community-based surveys along major drug trafficking routes. *Harm Reduction Journal* 2008; **5 (no pagination)**(29).
139. Lee KCK, Lim WWL, Lee SS. High prevalence of HCV in a cohort of injectors on methadone substitution treatment. *Journal of Clinical Virology* 2008; **41**(4): 297-300.
140. Mateu-Gelabert P, Harris S, Berbesi D, et al. Heroin use and injection risk behaviors in Colombia: Implications for HIV/AIDS prevention. *Substance Use & Misuse* 2016; **51**(2): 230-40.

141. Berbesi D, Segura A, Cardona D, Agudelo A. Factors associated with syringe exchange among injection drug users in Colombia. *Journal of Substance Use* 2016: 1-7.
142. Berbesi Fernández D, Montoya Vélez L, Segura Cardona A, Mateu-Gelabert P. Estudio de Prevalencia de VIH y Comportamientos de Riesgo Asociados, en Usuarios de Drogas por Via Inyectada (UDI) en Medellin y Pereira, 2011.
143. Handanagic S, Bozicevic I, Civljak M, et al. HIV and hepatitis C prevalence, and related risk behaviours among people who inject drugs in three cities in Croatia: Findings from respondent-driven sampling surveys. *International Journal of Drug Policy* 2016; **32**: 57-63.
144. Kosanovic ML, Kolaric B. Risk behaviour and risk for HIV infection in population of injecting drug users. (Rizicno ponasanje intravenskih korisnika droge i rizik zarazavanja HIV-om). *Infektoloski Glasnik* 2009; **29**(1): 5-11.
145. Kolarić B, Štajduhar D, Gajnik D, Rukavina T, Wiessing L. Seroprevalence of Blood-Borne Infections and Population Size Estimates in a Population of Injecting Drug Users in Croatia. *Central European Journal of Public Health* 2010; **18**(2): 104-9.
146. Kolaric B. Croatia: still a low-level HIV epidemic?--seroprevalence study. *Collegium Antropologicum* 2011; **35**(3): 861-5.
147. Cavlek TV, Maric J, Katicic L, Kolaric B. Hepatitis C virus antibody status, sociodemographic characteristics, and risk behaviour among injecting drug users in Croatia. *Central European Journal of Public Health* 2011; **19**(1): 26-9.
148. Reitox National Focal Point. 2011 National Report (2010 data) to the EMCDDA. Cyprus: New developments, trends and in-depth information on selected issues, 2012.
149. Demetriou VL, van de Vijver DAMC, Hezka J, Kostrikis LG, Cyprus Ivdu Network. Hepatitis C infection among intravenous drug users attending therapy programs in Cyprus. *Journal of Medical Virology* 2010; **82**(2): 263-70.
150. Chlibek R, Smetana J, Sosovickova R, et al. Prevalence of hepatitis C virus in adult population in the Czech Republic - Time for birth cohort screening. *PloS one* 2017; **12 (4) (no pagination)**(e0175525).
151. Reitox National Focal Point, Mravcik V, Pesek R, et al. 2010 National Report (2009 data) to the EMCDDA. The Czech Republic New Development, Trends and in-depth information on selected issues, 2011.
152. Bouscaillou J, Evanno J, Prouté M, et al. Prevalence and factors associated with HIV and tuberculosis in people who use drugs in Abidjan, Ivory Coast. *International Journal of Drug Policy* 2016: 8.
153. USAID. Rapport Annuel 2014, 2015.

154. Madsen LW, Fabricius T, Moessner BK, Birkemose I, Skamling M, Christensen PB. Depression is frequent among drug users but not related to hepatitis c infection. *Hepatology* 2011; **54**: 1187A.
155. Kinnard EN, Howe CJ, Kerr T, Skjodt Hass V, Marshall BD. Self-reported changes in drug use behaviors and syringe disposal methods following the opening of a supervised injecting facility in Copenhagen, Denmark. *Harm Reduction Journal* 2014; **11**: 29.
156. Axelsson A, Soholm H, Dalsgaard M, et al. Echocardiographic findings suggestive of infective endocarditis in asymptomatic danish injection drug users attending urban injection facilities. *American Journal of Cardiology* 2014; **114**(1): 100-4.
157. Wang M, Shen J, Deng Y, et al. Association of higher-risk alcohol consumption with injecting paraphernalia sharing behaviours in intravenous drug users. *American Journal of Drug & Alcohol Abuse* 2014; **40**(2): 137-42.
158. Cherry S, Williams H, Oyefeso A, Bennett J. Injecting other users: A pilot study in an area of high prevalence of drug-related deaths. *Journal of Substance Use* 2009; **14**(5): 289-94.
159. Bishton E, Oluboyede F, Grylls E, Woods L, Thomas S. Screening for Hepatitis C in injecting and ex-injecting drug users in North East Essex. *Public Health* 2014; **128**(11): 1036-8.
160. Rice P, Abou-Saleh MT. Detecting antibodies to hepatitis c in injecting drug users: A comparative Study between saliva, serum, and dried blood spot tests. *Addictive Disorders and their Treatment* 2012; **11**(2): 76-83.
161. Hope VD, Hickman M, Ngui SL, et al. Measuring the incidence, prevalence and genetic relatedness of hepatitis C infections among a community recruited sample of injecting drug users, using dried blood spots. *Journal of Viral Hepatitis* 2011; **18**(4): 262-70.
162. Hickman M, Hope V, Coleman B, et al. Assessing IDU prevalence and health consequences (HCV, overdose and drug-related mortality) in a primary care trust: implications for public health action. *Journal of Public Health* 2009; **31**(3): 374-82.
163. Reitox National Focal Point, Talu A, Abel-Ollo K, et al. 2012 National Report (2011 data) to EMCDDA. Estonia: New developments, trends and in-depth information on selected issues: Estonian National Institute for Health Development

Estonian Drug Monitoring Centre/REITOX Estonian Drug Information Centre, 2013.
164. Reitox National Focal Point, Abel-Ollo K, Talu A, et al. 2011 National Report (2010 data) to the EMCDDA. Estonia: New developments, trends and in-depth information on selected issues, 2012.
165. Jogeda EL, Avi R, Pauskar M, et al. Human T-lymphotropic virus types 1 and 2 are rare among intravenous drug users in Eastern Europe. *Infection, Genetics and Evolution* 2016; **43**: 83-5.

166. Ruutel K, Karnite A, Talu A, et al. Prevalence of IGRA-positivity and risk factors for tuberculosis among injecting drug users in Estonia and Latvia. *International Journal of Drug Policy* 2014; **25**(1): 175-8.
167. Ruutel K, Parker RD, Sobolev I, Loit HM. Tuberculosis knowledge among injecting drug users visiting syringe exchange programme in Tallinn, Estonia. *Central European Journal of Public Health* 2012; **20**(4): 248-51.
168. Uuskula A, Raag M, Abel-Ollo K, et al. HSV-2 seroprevalence among current injection drug users in Estonia. *Sexually Transmitted Infections Conference: STI and AIDS World Congress* 2013; **89**(no pagination).
169. Uuskula A, McMahon JM, Raag M, et al. Emergent properties of HIV risk among injection drug users in Tallinn, Estonia: synthesis of individual and neighbourhood-level factors. *Sexually Transmitted Infections* 2010; **86 Suppl 3**: iii79-84.
170. Uuskula A, Kals M, Rajaleid K, et al. High-prevalence and high-estimated incidence of HIV infection among new injecting drug users in Estonia: need for large scale prevention programs. *Journal of Public Health* 2008; **30**(2): 119-25.
171. Talu A, Rajaleid K, Abel-Ollo K, et al. HIV infection and risk behaviour of primary fentanyl and amphetamine injectors in Tallinn, Estonia: implications for intervention. *International Journal of Drug Policy* 2010; **21**(1): 56-63.
172. Ruutel K, Ustina V, Parker RD. Piloting HIV rapid testing in community-based settings in Estonia. *Scandinavian Journal of Public Health* 2012; **40**(7): 629-33.
173. Curatio International Foundation, Public Union Bemoni. HIV risk and prevention behaviors among People Who Inject Drugs in seven cities of Georgia 2015. 2015.
174. Curatio International Foundation, Public Union Bemoni. HIV risk and prevention behaviours among People Who Inject Drugs in six cities of Georgia Bio-behavioral surveillance survey in Tbilisi, Batumi, Zugdidi, Telavi, Gori, Kutaisi in 2012. 2012.
175. Curatio International Foundation, Public Union Bemoni. Bio-behavioral surveillance surveys among injecting drug users in Georgia (Tbilisi, Batumi, Zugdidi, Telavi, Gori, 2008 - 2009). 2009.
176. Otiashvili D, Zabransky T, Kirtadze I, Piralishvili G, Chavchanidze M, Miovsky M. Why do the clients of Georgian needle exchange programmes inject buprenorphine? *European Addiction Research* 2010; **16**(1): 1-8.
177. Chikovani I, Bozicevic I, Goguadze K, Rukhadze N, Gotsadze G. Unsafe injection and sexual risk behavior among injecting drug users in Georgia. *Journal of Urban Health* 2011; **88**(4): 736-48.

178. Bouscaillou J, Champagnat J, Luhmann N, et al. Hepatitis C among people who inject drugs in Tbilisi, Georgia: an urgent need for prevention and treatment. *International Journal of Drug Policy* 2014; **25**(5): 871-8.
179. Wenz B, Nielsen S, Gassowski M, et al. High variability of HIV and HCV seroprevalence and risk behaviours among people who inject drugs: results from a cross-sectional study using respondent-driven sampling in eight German cities (2011-14). *BMC Public Health* 2016; **16**(927): 14.
180. Muller MC, Pichler M, Martin G, et al. Burden of disease and level of patient's medical care in substitution treatment for opiates. [German] Krankheitslast und Versorgungsniveau bei opioidsubstituierten Patienten. *Medizinische Klinik* 2009; **104**(12): 913-7.
181. Thane K, Wickert C, Verthein U. Consumption patterns, risk behaviour and service needs in Germany's open drug scenes. *Sucht: Zeitschrift fur Wissenschaft und Praxis* 2011; **57**(2): 141-9.
182. Stover H, Schaffer D. Smoke It! Promoting a change of opiate consumption pattern-From injecting to inhaling. *Harm Reduction Journal* 2014; **11**: 18.
183. Schulte B, Schmidt CS, Strada L, et al. Non-prescribed use of opioid substitution medication: Patterns and trends in sub-populations of opioid users in Germany. *International Journal of Drug Policy* 2016; **29**: 57-65.
184. Backmund M, Meyer K, Schutz C, Reimer J. Factors associated with suicide attempts among injection drug users. *Substance Use & Misuse* 2011; **46**(12): 1553-9.
185. Reimer J, Verthein U, Karow A, Schafer I, Naber D, Haasen C. Physical and mental health in severe opioid-dependent patients within a randomized controlled maintenance treatment trial. *Addiction* 2011; **106**(9): 1647-55.
186. University of Ghana School of Public Health, Adanu R, Quaye S, et al. Understanding the Social, Economic and Behavioral Vulnerability to HIV of People Who Inject Drugs (PWID) in Accra/Tema and Takoradi, Ghana: University of Ghana School of Public Health,, 2016.
187. Reitox National Focal Point, Terzidou M, Antaraki A, et al. 2011 National Report (2010 data) to the EMCDDA. Greece: New developments, trends and in-depth information on selected issues, 2012.
188. Reitox National Focal Point, Terzidou M, Antaraki A, et al. 2012 National Report (2011 data) to the EMCDDA. Greece: New Development, Trends and in-depth information on selected issues, 2013.
189. Reitox National Focal Point, Terzidou M, Antaraki A, et al. 2014 National Report (2013 data) to the EMCDDA. Greece: New Development, Trends, 2015.
190. Antaraki A, Bafi I, Fotiou A, et al. Greece: New Development, Trends and in-depth information on selected issues. 2009 National Report to the EMCDDA, 2010.
191. Zavitsanou A, Malliori M, Sypsa V, et al. Seroepidemiology of human herpesvirus 8 (HHV-8) infection in injecting drug users. *Epidemiology and Infection* 2010; **138**(3): 403-8.

192. Hatzakis A, Sypsa V, Paraskevis D, et al. Design and baseline findings of a large-scale rapid response to an HIV outbreak in people who inject drugs in Athens, Greece: the Aristotle programme. *Addiction* 2015; **110**(9): 1453-67.
193. Tsang MA, Schneider JA, Sypsa V, et al. Network Characteristics of People Who Inject Drugs Within a New HIV Epidemic Following Austerity in Athens, Greece. *Journal of Acquired Immune Deficiency Syndromes: JAIDS* 2015; **69**(4): 499-508.
194. Tarjan A, Dudas M, Wiessing L, et al. HCV prevalence and risk behaviours among injectors of new psychoactive substances in a risk environment in Hungary-An expanding public health burden. *International Journal of Drug Policy* 2017; **41**: 1-7.
195. Reitox National Focal Point. 2011 National Report (2010 data) to the EMCDDA. Hungary: New developments, trends and in-depth information on selected issues, 2012.
196. Reitox National Focal Point, Karoly B, Tamas C, et al. 2010 National Report to the EMCDDA. "Hungary" New developments, trends and in-depth information on selected issues., 2011.
197. Reitox National Focal Point. 2014 National Report (2013 data) to the EMCDDA. Hungary: New Development, Trends, 2015.
198. Tamas C, Agnes C, Zsuzsanna E, et al. "Hungary": New developments, trends and in-depth information on selected issues. 2009 National Report to the EMCDDA, 2010.
199. National Centre for Epidemiology Hungary. A 2014 évben regisztrált HIV-fertőzöttek, és az újonnan bejelentett AIDS betegek korcsoportok szerint. 2015.
200. Marvanykovi F, Melles K, Racz J. Sex and drugs: The correlations of injecting drug users' risk perception and behavioral patterns. *Substance Use and Misuse* 2009; **44**(4): 569-77.
201. Tarjan A, Dudas M, Gyarmathy V, Rusvai E, Treso B, Csohan A. Emerging risks due to new injecting patterns in Hungary during austerity times. *Substance Use & Misuse* 2015; **50**(7): 848-58.
202. Csak R, Demetrovics Z, Racz J. Transition to injecting 3,4-methylene-dioxy-pyrovalerone (MDPV) among needle exchange program participants in Hungary. *Journal of Psychopharmacology* 2013; **27**(6): 559-63.
203. Gyarmathy VA, Neaigus A, Ujhelyi E. Vulnerability to drug-related infections and co-infections among injecting drug users in Budapest, Hungary. *European Journal of Public Health* 2009; **19**(3): 260-5.
204. Bjarnadottir GD, Haraldsson HM, Rafnar BO, et al. Prevalent intravenous abuse of methylphenidate among treatment-seeking patients with substance abuse disorders: A descriptive population-based study. *Journal of Addiction Medicine* 2015; **9**(3): 188-94.

205. Collazo EM. Healthcare Service Access, Sexual Aggression Experiences, and HIV-Related Risk Behaviors among Puerto Rican Female Intravenous Drug Users. ProQuest LLC: Indiana University; 2015.
206. Armstrong G, Nuken A, Samson L, Singh S, Jorm AF, Kermode M. Quality of life, depression, anxiety and suicidal ideation among men who inject drugs in Delhi, India. *BMC Psychiatry* 2013; **13**(151): 11.
207. Sabri B, McFall AM, Solomon SS, et al. Gender Differences in Factors Related to HIV Risk Behaviors among People Who Inject Drugs in North-East India. *PLoS ONE [Electronic Resource]* 2017; **12**(1).
208. FHI360. India: IBBA 2009-2010. 2011.
209. India: NACO. India: National Integrated Biological and Behavioural Surveillance (IBBS) 2014-15. 2015.
210. Mahanta J, Medhi GK, Paranjape RS, et al. Injecting and sexual risk behaviours, sexually transmitted infections and HIV prevalence in injecting drug users in three states in India. *Aids* 2008; **22**(SUPPL. 5): S59-S68.
211. Medhi GK, Mahanta J, Paranjape RS, et al. Factors associated with ever HIV testing among injecting drug users (IDUs) in two HIV high prevalent States of India. *Indian Journal of Medical Research, Supplement* 2012; **136**(SUPPL): 64-71.
212. Solomon SS, Desai M, Srikrishnan AK, et al. The profile of injection drug users in Chennai, India: identification of risk behaviours and implications for interventions. *Substance Use & Misuse* 2010; **45**(3): 354-67.
213. Suohu K, Humtsoe C, Saggurti N, Sabarwal S, Mahapatra B, Kermode M. Understanding the association between injecting and sexual risk behaviors of injecting drug users in Manipur and Nagaland, India. *Harm Reduction Journal* 2012; **9**: 40.
214. Tun W, Sebastian MP, Sharma V, et al. Strategies for recruiting injection drug users for HIV prevention services in Delhi, India. *Harm Reduction Journal* 2013; **10**: 16.
215. Sarin E, Kerrigan D. The impact of human rights violations and perceptions of discrimination on health service utilization among injection drug users in Delhi, India. *Substance Use & Misuse* 2012; **47**(3): 230-43.
216. Sarin E, Singh B, Samson L, Sweat M. Suicidal ideation and HIV risk behaviors among a cohort of injecting drug users in New Delhi, India. *Substance Abuse Treatment, Prevention, and Policy* 2013; **8**: 2.
217. Sarna A, Tun W, Bhattacharya A, Lewis D, Singh YS, Apicella L. Assessment of unsafe injection practices and sexual behaviors among male injecting drug users in two urban cities of India using

respondent driven sampling. *Southeast Asian Journal of Tropical Medicine & Public Health* 2012; **43**(3): 652-67.

218. Kermode M, Armstrong G, Medhi GK, Humtsoe C, Langkham B, Mahanta J. Sexual behaviours of men who inject drugs in Northeast India. *Harm Reduction Journal* 2015; **12**: 4.

219. Kumar S, Garg PD, Ambekar A, Bala N. Opioid substitution therapy under national AIDS control programme at GMC, Amritsar: Profile of patients recruited in first 1 year. *Indian Journal of Psychiatry* 2013; **55**: S127.

220. Ambekar A, Rao R, Mishra AK, Agrawal A. Type of opioids injected: does it matter? A multicentric cross-sectional study of people who inject drugs. *Drug & Alcohol Review* 2015; **34**(1): 97-104.

221. Armstrong G, Humtsoe C, Kermode M. HIV risk behaviours among injecting drug users in Northeast India following scale-up of a targeted HIV prevention programme. *BMC Public Health* 2011; **11 Suppl 6**: S9.

222. Biswas K, Arumugam V, Sharma C, Rakesh S, Robertson J. Getting high, getting laid: Injecting practices and sexual behaviour of people who inject drugs (PWID) in three Indian states (findings from the Hridaya baseline study). *Journal of the International AIDS Society* 2012; **15**: 195-6.

223. Ghosh I, Ghosh P, Bharti AC, Mandal R, Biswas J, Basu P. Prevalence of human papillomavirus and co-existent sexually transmitted infections among female sex workers, men having sex with men and injectable drug abusers from eastern India. *Asian Pacific Journal of Cancer Prevention: Apjcp* 2012; **13**(3): 799-802.

224. Chalana H, Singh H, Sachdeva JK, Sharma S. Seroprevalence of human immunodeficiency virus, hepatitis B surface antigen, and hepatitis C in substance dependents admitted in a tertiary hospital at Amritsar, India. *Asian Journal of Psychiatry* 2013; **6**(6): 552-5.

225. Sarkar K, Das SSj, Pal R, Bal B, Madhusudan P, Chakraborti S. HIV infection and host genetic mutation among injecting drug-users of northeastern states of India. *Journal of Health, Population & Nutrition* 2010; **28**(2): 130-6.

226. Sharma AK, Bn S, Basu D. HCV and HIV co-infection and related risks in injecting drug users from a superspeciality centre. *Hepatology* 2014; **60**: 933A.

227. Shukla A, Sharma A. Seroprevalence of hepatitis B, hepatitis C and human immunodeficiency viruses amongst injecting drug users in Mumbai,India. *Journal of Clinical and Diagnostic Research* 2014; **8**(6).

228. Solomon SS, Mehta SH, Srikrishnan AK, et al. Burden of hepatitis C virus disease and access to hepatitis C virus services in people who inject drugs in India: a cross-sectional study. *The Lancet Infectious Diseases* 2015; **15**(1): 36-45.

229. Solomon SS, Srikrishnan AK, McFall AM, et al. Burden of liver disease among community-based people who inject drugs (PWID) in Chennai, India. *PLoS ONE [Electronic Resource]* 2016; **11 (1) (no pagination)**(e0147879).

230. Solomon SS, Srikrishnan AK, Mehta SH, et al. High prevalence of HIV, HIV/hepatitis C Virus coinfection, and risk behaviors among injection drug users in Chennai, India: A cause for concern. *Journal of Acquired Immune Deficiency Syndromes* 2008; **49**(3): 327-32.

231. Saraswati LR, Sarna A, Sebastian MP, et al. HIV, Hepatitis B and C among people who inject drugs: high prevalence of HIV and Hepatitis C RNA positive infections observed in Delhi, India. *BMC Public Health* 2015; **15**: 726.

232. Panda S, Roy T, Pahari S, et al. Alarming epidemics of human immunodeficiency virus and hepatitis C virus among injection drug users in the northwestern bordering state of Punjab, India: Prevalence and correlates. *International Journal of STD and AIDS* 2014; **25**(8): 596-606.

233. Mahanta J, Borkakoty B, Das HK, Chelleng PK. The risk of HIV and HCV infections among injection drug users in northeast India. *AIDS Care* 2009; **21**(11): 1420-4.

234. Lucas GM, Solomon SS, Srikrishnan AK, et al. High HIV burden among people who inject drugs in 15 Indian cities. *AIDS* 2015; **29**(5): 619-28.

235. Goswami P, Medhi GK, Armstrong G, et al. An assessment of an HIV prevention intervention among people who inject drugs in the states of Manipur and Nagaland, India. *International Journal of Drug Policy* 2014; **25**(5): 853-64.

236. Altaf A, Saleem N, Abbas S, Muzaffar R. High prevalence of HIV infection among injection drug users (IDUs) in Hyderabad and Sukkur, Pakistan. *JPMA - Journal of the Pakistan Medical Association* 2009; **59**(3): 136-40.

237. National AIDS Comission, HIV Cooperation Program for Indonesia. Injecting Drug User Behaviour and Service Satisfaction Survey. 2013.

238. Persaudaraan Korban Napza Indonesia (PKNI). Peer-Driven Intervention on Hepatitis C Testing and Treatment Literacy among People Who Inject Drugs in Jakarta, Indonesia. 2014.

239. National AIDS Comission Indonesia. Rapid Behavioral Survey among Injecting Drugs Users. 2010.

240. AIDS Data Hub for Asia-Pacific. Integrated Biological and Behavioural Survey Indonesia. 2011.

241. Morineau G, Bollen LJ, Syafitri RI, Nurjannah N, Mustikawati DE, Magnani R. HIV prevalence and risk behaviours among injecting drug users in six Indonesian cities implications for future HIV prevention programs. *Harm Reduction Journal* 2012; **9**: 37.

242. Iskandar S, Basar D, Hidayat T, et al. High risk behavior for HIV transmission among former injecting drug users: a survey from Indonesia. *BMC Public Health* 2010; **10**: 472.

243. Afriandi I, Siregar AY, Meheus F, et al. Costs of hospital-based methadone maintenance treatment in HIV/AIDS control among injecting drug users in Indonesia. *Health Policy* 2010; **95**(1): 69-73.
244. Achmad YM, Istiqomah AN, Iskandar S, Wisaksana R, van Crevel R, Hidayat T. Integration of methadone maintenance treatment and HIV care for injecting drug users: a cohort study in Bandung, Indonesia. *Acta Medica Indonesiana* 2009; **41 Suppl 1**: 23-7.
245. Noroozi A, Mirzazadeh A, Hajebi A, et al. Comparing profile of people who inject drugs (PWID) accessing different types of needle and syringe programs or secondary distribution in Kermanshah, Iran. *Journal of Substance Use* 2016: 1-6.
246. Kakavand-Ghalehnoei R, Shoja Z, Najafi A, et al. Prevalence of human herpesvirus-8 among HIV-infected patients, intravenous drug users and the general population in Iran. *Sexual Health* 2016; **13**(3): 295-8.
247. Zamani S, Radfar R, Nematollahi P, et al. Prevalence of HIV/HCV/HBV infections and drug-related risk behaviours amongst IDUs recruited through peer-driven sampling in Iran. *International Journal of Drug Policy* 2010; **21**(6): 493-500.
248. Zamani S, Vazirian M, Nassirimanesh B, et al. Needle and syringe sharing practices among injecting drug users in Tehran: a comparison of two neighborhoods, one with and one without a needle and syringe program. *AIDS & Behavior* 2010; **14**(4): 885-90.
249. Maghsoudi A, Baneshi MR, Neydavoodi M, Haghdoost A. Network scale-up correction factors for population size estimation of people who inject drugs and female sex workers in Iran. 2014; **9**(11): e110917.
250. Mamani M, Majzoobi MM, Torabian S, Mihan R, Alizadeh K. Latent and active tuberculosis: Evaluation of injecting drug users. *Iranian Red Crescent Medical Journal* 2013; **15**(9): 775-9.
251. Mehrjerdi ZA, Abarashi Z, Noroozi A, Arshad L, Zarghami M. Correlates of shared methamphetamine injection among methamphetamine-injecting treatment seekers: The first report from Iran. *International Journal of STD and AIDS* 2014; **25**(6): 420-7.
252. Sahebi L, Abadi MAJ, Mousavi SH, Khalili M, Seyedi M. Relationship between psychiatric distress and criminal history among intravenous drug abusers in Iran. *Iranian Journal of Psychiatry and Behavioral Sciences* 2015; **9**(2): 37-42.
253. Sharif M, Sherif A, Sayyah M. Frequency of HBV, HCV and HIV infections among hospitalized injecting drug users in Kashan. *Indian Journal of Sexually Transmitted Diseases* 2009; **30**(1): 28-30.
254. Abdollahi Z, Taghizadeh F, Hamzehgardeshi Z, Bahramzad O. Relationship between addiction relapse and self-efficacy rates in injection drug users referred to Maintenance Therapy Center of Sari, 1391. *Global Journal of Health Science* 2014; **6**(3): 138-44.

255. Assari S, Ahmadi K, Rezazade M. Socio-economic status determines risk of receptive syringe sharing behaviors among Iranian drug injectors; a national study. *Frontiers in Psychiatry* 2015; **6 (MAR) (no pagination)**(194).
256. Assari S, Yarmohamadivasel M, Lankarani MM, et al. Having multiple sexual partners among Iranian intra-venous drug users. *Frontiers in Psychiatry* 2014; **5**: 125.
257. Kandelouei T, Hosseini SM, Gachkar L, Keyvani H, Davoodbeglou F, Vaezjalali M. Reduction in prevalence of hepatitis B surface antigen among intravenous drug users in Tehran drop-in-centers. *Archives of Clinical Infectious Diseases* 2013; **8 (2) (no pagination)**(e15531).
258. Nokhodian Z, Meshkati M, Adibi P, et al. Hepatitis C screening in isfahan drop in centers: An experience description. *International Journal of Preventive Medicine* 2012; **3**(Suppl1): S131-S8.
259. Mir-Nasseri M, Poustchi H, Nasseri-Moghadam S, Mohammadkhani A, Malekzadeh R. Hepatitis C seroprevalence among intravenous drug users in Tehran. *Journal of gastroenterology and hepatology* 2013; **28**: 412.
260. Amin-Esmaeili M, Rahimi-Movaghar A, Razaghi EM, Baghestani AR, Jafari S. Factors correlated with hepatitis C and B virus infections among injecting drug users in Tehran, IR Iran. *Hepatitis Monthly* 2012; **12**(1): 23-31.
261. Nobari RF, Meshkati M, Ataei B, et al. Identification of patients with hepatitis c virus infection in persons with background of intravenous drug use: The first community announcementbased study from Iran. *Hepatology International* 2012; **7**: S406-S7.
262. Alavian SM, Mirahmadizadeh A, Javanbakht M, et al. Effectiveness of methadone maintenance treatment in prevention of hepatitis C virus transmission among injecting drug users. *Hepatitis Monthly* 2013; **13**(8): 9.
263. Rahimi-Movaghar A, Razaghi EM, Sahimi-Izadian E, Amin-Esmaeili M. HIV, hepatitis C virus, and hepatitis B virus co-infections among injecting drug users in Tehran, Iran. *International Journal of Infectious Diseases* 2010; **14**(1): e28-33.
264. Ramezani A, Amirmoezi R, Volk JE, et al. HCV, HBV, and HIV seroprevalence, coinfections, and related behaviors among male injection drug users in Arak, Iran. *AIDS Care* 2014; **26**(9): 1122-6.
265. Khajehkazemi R, Osooli M, Sajadi L, et al. HIV prevalence and risk behaviours among people who inject drugs in Iran: the 2010 National Surveillance Survey. *Sexually Transmitted Infections* 2013; **89 Suppl 3**: iii29-32.
266. Malekinejad M, Mohraz M, Razani N, et al. High HIV prevalence in a respondent-driven sampling survey of injection drug users in Tehran, Iran. *AIDS & Behavior* 2015; **19**(3): 440-9.
267. Momen-Heravi M, Afzali H, Moosavipanah H. Prevalence of anti HIV, antiHCV and, HBSAG positive among injection drug users in Kashan-Iran. *Journal of Clinical Immunology* 2012; **32**: S391.

268. Hosseini M, SeyedAlinaghi SA, Kheirandish P, et al. Prevalence and correlates of co-infection with human immunodeficiency virus and hepatitis C virus in male injection drug users in Iran. *Archives of Iranian Medicine* 2010; **13**(4): 318-23.
269. Jennings CJ. Re-establishing Contact: A profile of clients attending the Health Promotion Unit - Needle Exchange at Merchants Quay Ireland, 2013.
270. Maloney S, Keenan E, Geoghegan N. What are the risk factors for soft tissue abscess development among injecting drug users? *Nursing Times* 2010; **106**(23): 21-4.
271. WHO. HIV Bio-Behavioral Survey among Injecting Drug Users in the East Jerusalem Governorate. 2010.
272. Napoli C, Tafuri S, Pignataro N, Tedesco G, Santa Maria A, Quarto M. Risk factors for HBV/HIV/HCV in drug addicts: A survey of attendees of a Department of Pathological Dependence. *Journal of Preventive Medicine and Hygiene* 2010; **51**(3): 101-4.
273. Pavarin RM. Mortality risk in intravenous drug users in Bologna and its determining factors. Results of a longitudinal study. *Epidemiologia e Prevenzione* 2008; **32**(2): 99-107.
274. Camoni L, Federico B, Capelli G, et al. Few Italian drug users undergo HIV testing. *AIDS & Behavior* 2011; **15**(4): 711-7.
275. Nosotti L, Fagetti R, Rocchi L, et al. Prevalence of HCV infection and adherence to DOT therapy in Italian and non-Italian IV drug users in Rome, Italy. *Heroin Addiction and Related Clinical Problems* 2014; **16**(1): 41-4.
276. Population Services International. HIV and TB TRaC study evaluating risk behaviors associated with HIV transmission and utilization of HIV prevention and HIV/TB co-infection prevention among IDUs. Round one. 2010.
277. Singh K, Brodish P, Mbai F, et al. A venue-based approach to reaching MSM, IDUs and the general population with VCT: A three study site in Kenya. *AIDS and Behavior* 2012; **16**(4): 818-28.
278. Syvertsen JL, Agot K, Ohaga S, et al. Evidence of injection drug use in Kisumu, Kenya: Implications for HIV prevention. *Drug and Alcohol Dependence* 2015; **151**: 262-6.
279. Bhattacharjee P, McClarty LM, Musyoki H, et al. Monitoring HIV prevention programme outcomes among key populations in Kenya: Findings from a national survey. *PloS one* 2015; **10 (8) (no pagination)**(e0137007).
280. Brodish P, Singh K, Rinyuri A, et al. Evidence of high-risk sexual behaviors among injection drug users in the Kenya PLACE study. *Drug and Alcohol Dependence* 2011; **119**(1-2): 138-41.
281. Tun W, Sheehy M, Broz D, et al. HIV and STI prevalence and injection behaviors among people who inject drugs in Nairobi: results from a 2011 bio-behavioral study using respondent-driven sampling. *AIDS and Behavior* 2015; **19 Suppl 1**: S24-35.

282. Mwatelah RS, Lwembe RM, Osman S, et al. Co-infection burden of hepatitis C virus and human immunodeficiency virus among injecting heroin users at the kenyan coast. *PLoS ONE [Electronic Resource]* 2015; **10 (7) (no pagination)**(e0132287).
283. Kurth AE, Cleland CM, Des Jarlais DC, et al. HIV Prevalence, Estimated Incidence, and Risk Behaviors Among People Who Inject Drugs in Kenya. *Journal of Acquired Immune Deficiency Syndromes: JAIDS* 2015; **70**(4): 420-7.
284. Yun H, Kim D, Kim S, et al. High prevalence of HBV and HCV infection among intravenous drug users in Korea. *Journal of Medical Virology* 2008; **80**(9): 1570-5.
285. Min JA, Yoon Y, Lee HJ, et al. Prevalence and associated clinical characteristics of hepatitis B, C, and HIV infections among injecting drug users in Korea. *Journal of Medical Virology* 2013; **85**(4): 575-82.
286. Rosenkranz M, Kerimi N, Takenova M, et al. Assessment of health services for people who use drugs in Central Asia: findings of a quantitative survey in Kazakhstan and Kyrgyzstan. *Harm Reduction Journal* 2016; **13**: 3.
287. Chokmorova U, Ismailova A, Bubusara S, et al. IBBS 2013 Report. 2013.
288. Phimphachanh C. Rapid Assessment and Response to Drug Use and Injecting Drug Use in Huaphanh and Phongsaly Provinces, Lao PDR. 2011.
289. Reitox National Focal Point, Pugule I, Trapencieris M, et al. 2012 National Report (2011 data) to the EMCDDA. Latvia: New developments, trends and in-depth information on selected issues. 2013.
290. Expanding Network for Comprehensive and Coordinated Action on HIV/AIDS prevention among IDUs and Bridging Population (ENCAP). Prevalence of HIV and other infections and risk behaviour among Injecting Drug Users in Latvia, Lithuania and Estonia in 2007, 2009. 2009.
291. Merabi Z, Naja WJ, Soufia M, et al. Intranasal heroin use - an emerging trend in Lebanon: A single institution study presenting sociodemographic profiles of intranasal versus intravenous users. *Journal of Substance Use* 2016: 1-6.
292. Mahfoud Z, Afifi R, Ramia S, et al. HIV/AIDS among female sex workers, injecting drug users and men who have sex with men in Lebanon: Results of the first biobehavioral surveys. *Aids* 2010; **24**(SUPPL. 2): S45-S54.
293. Mirzoyan L, Berendes S, Jeffery C, et al. New evidence on the HIV epidemic in Libya: Why countries must implement prevention programs among people who inject drugs. *JAIDS Journal of Acquired Immune Deficiency Syndromes* 2013; **62**(5): 577-83.
294. Gyarmathy VA, Caplinskiene I, Caplinskas S, Latkin CA. Social network structure and HIV infection among injecting drug users in Lithuania: gatekeepers as bridges of infection. *AIDS & Behavior* 2014; **18**(3): 505-10.

295. Petrushevska T, Stefanovska VV. Patients on Opioid Substitution Treatment in the Republic of Macedonia: What do Treatment Demand Data Tell Us? *Macedonian Journal of Medical Sciences* 2014; **2**(2): 335-43.
296. Kiprijanovska S, Davalieva K, Noveski P, Sukarova-Stefanovska E, Plaseska-Karanfilska D, Polenakovic M. Prevalence of hepatitis C virus genotypes in four risk groups in the Republic of Macedonia. *Journal of Viral Hepatitis* 2013; **20**: 18.
297. Système d'Information Multi-Sectorielle: Madagascar. Cartographie et estimation de la taille des personnes cibles les plus exposées aux risques du VIH/Sida. 2014.
298. African Development Bank Group. Etude comportementale et biologique chez les consommateurs de drogues injectables dans les zones urbaines à Madagascar-2012. 2012.
299. Chawarski MC, Vicknasingam B, Mazlan M, Schottenfeld RS. Lifetime ATS use and increased HIV risk among not-in-treatment opiate injectors in Malaysia. *Drug and Alcohol Dependence* 2012; **124**(1-2): 177-80.
300. Vicknasingam B, Narayanan S, Navaratnam V. The relative risk of HIV among IDUs not in treatment in Malaysia. *AIDS Care* 2009; **21**(8): 984-91.
301. Bazazi AR, Crawford F, Zelenev A, Heimer R, Kamarulzaman A, Altice FL. HIV prevalence among people who inject drugs in greater Kuala Lumpur recruited using respondent-driven sampling. *AIDS and behavior* 2015; **19**(12): 2347-57.
302. Republic of Maldives. Biological and Behavioral Survey (BBS) on HIV and AIDS 2008. 2008.
303. Mauritius: Ministry of Health and Quality of Life. Integrated Biological Behavioral Survey Among People Who Inject Drugs in Mauritius. 2011.
304. Johnston L, Saumtally A, Corceal S, Mahadoo I, Oodally F. High HIV and hepatitis C prevalence amongst injecting drug users in Mauritius: findings from a population size estimation and respondent driven sampling survey. *International Journal of Drug Policy* 2011; **22**(4): 252-8.
305. Baumbach JP, Foster LN, Mueller M, et al. Seroprevalence of select bloodborne pathogens and associated risk behaviors among injection drug users in the Paso del Norte region of the United States-Mexico border. *Harm Reduction Journal* 2008; **5**: 33.
306. Brouwer K, Lozada R, Cornelius W, et al. Deportation along the U.S.-Mexico border: Its relation to drug use patterns and accessing care. *Journal of Immigrant and Minority Health* 2009; **11**(1): 1-6.
307. Harvey-Vera AY, Gonzalez-Zuniga P, Vargas-Ojeda AC, et al. Risk of violence in drug rehabilitation centers: Perceptions of people who inject drugs in Tijuana, Mexico. *Substance Abuse Treatment, Prevention, and Policy* 2016; **11**: 5.

308. Rusch ML, Lozada R, Pollini RA, et al. Polydrug use among IDUs in Tijuana, Mexico: correlates of methamphetamine use and route of administration by gender. *Journal of Urban Health* 2009; **86**(5): 760-75.

309. Global Fund. Integrated Bio-Behavioural Study in key populations at higher risk: key indicators. 2013.

310. Bacak V, Lausevic D, Mugosa B, Vratnica Z, Terzic N. Hepatitis C virus infection and related risk factors among injection drug users in Montenegro. *European Addiction Research* 2013; **19**(2): 68-73.

311. Judd A, Rhodes T, Johnston LG, et al. Improving survey methods in sero-epidemiological studies of injecting drug users: a case example of two cross sectional surveys in Serbia and Montenegro. *BMC Infectious Diseases* 2009; **9**: 14.

312. Ministere de la Sante. Mise en Oeuvre de la Declaration Politique sur le VIH/Sida. Report to UNAIDS. 2014.

313. Toufik A. Enquêtes intégrées de surveillance bio-comportementale auprès des Usagers de Drogues Injectables à Tétouan. 2014.

314. Teodoro E, Boothe M, Baltazar C, et al. Urgent Need for Harm-Reduction Interventions in Mozambique: Results from the Integrated Bio-Behavioral Survey among People Who Inject Drugs. 2015.

315. National AIDS Program Myanmar. HIV Sentinel Sero-Surveillance Survey Report 2009. 2010.

316. WHO. HIV Sentinel Sero-Surveillance Survey Report 2010. 2011.

317. WHO. HIV Sentinel Sero-Surveillance Survey Report 2011. 2012.

318. Lou V, Johnston L, Soe PM. Myanmar Integrated Biological and Behavioral Surveillance Survey of People Who Inject Drugs, 2014.

319. WHO. HIV Sentinel Sero-Surveillance Survey Report 2014. 2015.

320. AIDS Data Hub. HIV Sentinel Sero-Surveillance Survey Report 2012. 2013.

321. Saw YM, Yasuoka J, Saw TN, Poudel KC, Tun S, Jimba M. What are the factors associated with HIV testing among male injecting and non-injecting drug users in Lashio, Myanmar: A cross-sectional study. *BMJ Open* 2013; **3 (6) (no pagination)**(e002747).

322. Swe LA, Rashid A. Prevalence of HIV and the risk behaviours among injecting drug users in Myanmar. *International Journal of Collaborative Research on Internal Medicine and Public Health* 2012; **4**(1): 56-70.

323. Loewinger G, Sharma B, Karki DK, Khatiwoda P, Kainee S, Poudel KC. Low knowledge and perceived Hepatitis C risk despite high risk behaviour among injection drug users in Kathmandu, Nepal. *International Journal of Drug Policy* 2016; **33**: 75-82.

324. Sharma V, Chamroonswasdi K, Srisorrachatr S. Rate of Adherence to and Factors Associated with Methadone Maintenance Treatment Program (Mmtp) Compliance among Injecting Drug Use

Patients in Nepal. *The Southeast Asian journal of tropical medicine and public health* 2016; **47**(2): 287-98.
325. Ministry of Health and Population Nepal. Integrated Biological and Behavioral Surveillance (IBBS) Survey among Injecting Drugs Users in Kathmandu Valley, Nepal. 2011.
326. Ministry of Health and Population Nepal. Integrated Biological and Behavioral Surveillance (IBBS) Survey among People who Inject Drugs (PWIDs) in Eastern Terai Highway Districts of Nepal. 2012.
327. Ministry of Health and Population Nepal. Integrated Biological and Behavioral Surveillance Survey (IBBS) among Male Injecting Drug Users (IDUs) in the Eastern Terai of Nepal Round IV. 2009.
328. Ministry of Health and Population Nepal. Integrated Biological and Behavioral Surveillance Survey (IBBS) among Injecting Drug Users in Kathmandu Valley 2009.
329. Ministry of Health and Population Nepal. Integrated Biological and Behavioral Surveillance Survey (IBBS) among Injecting Drug Users in Pokhara Valley. 2009.
330. Ministry of Health and Population Nepal. Integrated Biological and Behavioral Surveillance Survey (IBBS) among Male Injecting Drug Users (IDUs) in Western to Far-Western Terai of Nepal. 2009.
331. Nepal: National Centre for AIDS and STD Control. Mapping & Size Estimation of Most-At-Risk-Population in Nepal, 2011, Injecting Drug Users. 2011.
332. National Centre for AIDS and STD Control (NCASC). Integrated Biological and Behavioral Surveillance (IBBS) Survey among People Who Inject Drugs (PWID- Male) in the Eastern Terai Highway Districts (Jhapa, Morang and Sunsari) of Nepal. 2015.
333. National Centre for AIDS and STD Control (NCASC). Integrated Biological and Behavioral Surveillance (IBBS) Survey among People Who Inject Drugs (PWID) in Kathmandu Valley. 2015.
334. National Centre for AIDS and STD Control (NCASC). Integrated Biological and Behavioral Surveillance (IBBS) Survey among People Who Inject Drugs (PWIDs) in Pokhara Valley. 2015.
335. Poudel KC, Poudel-Tandukar K, Yasuoka J, Joshi AB, Jimba M. Correlates of sharing injection equipment among male injecting drug users in Kathmandu, Nepal. *International Journal of Drug Policy* 2010; **21**(6): 507-10.
336. Yadav DK. Burden of hepatitis 'C' among high risk people of HIV/IV drug users: A community based study from Eastern Nepal. *Sexually Transmitted Infections Conference: STI and AIDS World Congress* 2013; **89**(no pagination).
337. Ojha SP, Sigdel S, Verthien U, Khadga PK. HIV epidemiology in Nepal-"South Asian cocktail" a drug use pattern in Nepal and its correlation with spread of HIV. *Indian Journal of Psychiatry* 2014; **55**: S46-S7.

338. Kinkel HT, Karmacharya D, Shakya J, et al. Prevalence of HIV, hepatitis B and C infections and an assessment of HCV-genotypes and two IL28B SNPs among people who inject drugs in three regions of Nepal. *PLoS ONE [Electronic Resource]* 2015; **10 (8) (no pagination)**(e0134455).

339. Wilkins C, Prasad J, Wong K, Rychert M. Recent Trends in Illegal Drug Use in New Zealand, 2006-2014. Findings from the 2006, 2007, 2008, 2009, 2010, 2011, 2012, 2013 and 2014 Illicit Drug Monitoring System (IDMS). Massey University: Social and Health Outcomes Research and Evaluation College of Health, 2015.

340. Hay B, Henderson C, Maltby J, Canales JJ. Influence of peer-based needle exchange programs on mental health status in people who inject drugs: A nationwide New Zealand study. *Frontiers in Psychiatry* 2017; **7 (JAN) (no pagination)**(211).

341. Wilkins C, Prasad J, Wong K, Rychert M. Recent Trends in Illegal Drug Use in New Zealand, 2006-2013: Findings from the 2006, 2007, 2008, 2009, 2010, 2011, 2012 and 2013 Illicit Drug Monitoring System (IDMS). 2014.

342. Noller G, Leafe K. Brief analysis of the New Zealand population of people who inject drugs (PWID): Estimates of population size, prevalence of BBVs, demographics and risk behaviours, and service provision. 2016.

343. Judson G, Bird R, O'Connor P, et al. Drug injecting in patients in New Zealand methadone maintenance treatment programs: An anonymous survey. *Drug and Alcohol Review* 2010; **29**(1): 41-6.

344. Ministerio de Salud Nicaragua. Estudio de Vigilancia de Comportamiento Sexual y prevalencia del VIH y sífilis en poblaciones vulnerables y en mayor riesgo al VIH. 2014.

345. Federal Ministry of Health Nigeria. Nigeria IBBSS 2014.

346. Federal Ministry of Health (FMOH). Nigeria: HIV Integrated Biological and Behavioural Surveillance Survey (IBBSS). 2010.

347. Tun W, Vu L, Adebajo SB, et al. Population-based prevalence of hepatitis B and C virus, HIV, syphilis, gonorrhoea and chlamydia in male injection drug users in Lagos, Nigeria. *International Journal of STD & AIDS* 2013; **24**(8): 619-25.

348. Reitox National Focal Point. 2009 National Report (2008 data) to the EMCDDA. "Norway" New Developments, Trends and in-depth information on selected issues: Norwegian Institute for Alcohol and Drug Research - SIRUS, 2010.

349. Gjersing L, Bretteville-Jensen AL. Gender differences in mortality and risk factors in a 13-year cohort study of street-recruited injecting drug users. *BMC Public Health* 2014; **14**: 440.

350. Dalgard O, Egeland A, Ervik R, Vilimas K, Skaug K, Steen TW. Risk factors for hepatitis C among injecting drug users in Oslo. *Tidsskrift for Den Norske Laegeforening* 2009; **129**(2): 101-4.

351. Bretteville-Jensen AL, Lillehagen M, Gjersing L, Andreas JB. Illicit use of opioid substitution drugs: Prevalence, user characteristics, and the association with non-fatal overdoses. *Drug and Alcohol Dependence* 2015; **147**: 89-96.

352. Stulhofer A, Jwehan I, AbuRabie R. HIV and HCV prevalence and incarceration-related risks among injecting drug users in three West Bank governorates. *AIDS Care - Psychological and Socio-Medical Aspects of AIDS/HIV* 2016; **28**(9): 1159-65.

353. Chatty A, AbuRabie R, Dibeh S, et al. HIV Bio-Behavioural Suvery among Injecting Drug Users in the East Jerusalem Governorate, 2010, 2010.

354. Akhtar AM, Jamil M, Rehman A, Majeed S. Hepatitis-C virus infection among injecting drug users in Lahore, Pakistan: A cross sectional study. *Pakistan Journal of Medical Sciences* 2016; **32**(2): 373-8.

355. Raza MA, Sherwani RAK, Kamal S, Aftab M, Irfan M. Correlates of injection initiation among drug users in Punjab, Pakistan. *Pakistan Journal of Medical and Health Sciences* 2016; **10**(4): 1135-9.

356. AP Consultancies. Integrated Behavioural and Biological Surveillance, among Most at Risk Population IBBS Study – Punjab 2014 2014.

357. Emmanuel F. HIV Second Generation Surveillance in Pakistan - National Report Round IV. 2012.

358. National AIDS Control Program. HIV Second Generation Surveillance in Pakistan - National Report Round III. 2008.

359. Platt L, Vickerman P, Collumbien M, et al. Prevalence of HIV, HCV and sexually transmitted infections among injecting drug users in Rawalpindi and Abbottabad, Pakistan: evidence for an emerging injection-related HIV epidemic. *Sexually Transmitted Infections* 2009; **85 Suppl 2**: ii17-22.

360. Emmanuel F, Archibald C, Razaque A, Sandstrom P. Factors associated with an explosive HIV epidemic among injecting drug users in Sargodha, Pakistan. *Journal of Acquired Immune Deficiency Syndromes: JAIDS* 2009; **51**(1): 85-90.

361. Archibald CP, Shaw SY, Emmanuel F, et al. Geographical and temporal variation of injection drug users in Pakistan. *Sexually Transmitted Infections* 2013; **89 Suppl 2**: ii18-28.

362. HIV and AIDS Data Hub for Asia-Pacific. IHBSS Philippines. 2011.

363. HIV and AIDS Data Hub for Asia-Pacific. IHBSS Philippines. 2013.

364. AIDS Data Hub. IHBSS Philippines. 2009.

365. Reitox National Focal Point. 2014 National Drug Report (2013 data) to the EMCDDA. Poland: New Development, Trends and in-depth information on selected issues, 2015.

366. Reitox National Focal Point, Centrum Informacji o Nakotykach i Narkomanii - Reitox Polish National Focal Point, Malczewski A, et al. 2013 National Report (2012 data) to the EMCDDA. "Poland" New Development, Trends and in-depth information on selected issues, 2014.
367. Reitox National Focal Point, Malczewski A, Kidawa M, Struzik M, Strzelecka A. 2010 National Report (2009 data) to the EMCDDA. "Poland" New Development, Trends and in-depth information on selected issues, 2011.
368. Czerwinski M, McNutt LA, DeHovitz JA, Zielinski A, Rosinska M. Refining HIV Risk: The Modifying Effects of Youth, Gender and Education among People Who Inject Drugs in Poland. *PloS one* 2013; **8 (7) (no pagination)**(e68018).
369. Rosinska M, Sieroslawski J, Wiessing L. High regional variability of HIV, HCV and injecting risks among people who inject drugs in Poland: comparing a cross-sectional bio-behavioural study with case-based surveillance. *BMC Infectious Diseases* 2015; **15**: 83.
370. Calado RA, Rocha MR, Parreira R, Piedade J, Venenno T, Esteves A. Hepatitis C virus subtypes circulating among intravenous drug users in Lisbon, Portugal. *Journal of Medical Virology* 2011; **83**(4): 608-15.
371. Reyes-Ortiz VE. Social network correlates of HCV and HIV transmission risk behaviors among injecting drug users. *Dissertation Abstracts International: Section B: The Sciences and Engineering* 2016; **77**(5-B(E)): No Pagination Specified.
372. Zerden LDS, Marilis Lopez L, Lundgren LM. Needle sharing among Puerto Rican injection drug users in Puerto Rico and Massachusetts: place of birth and residence matter. *Substance Use & Misuse* 2010; **45**(10): 1605-22.
373. Abadie R, Welch-Lazoritz M, Gelpi-Acosta C, Reyes JC, Dombrowski K. Understanding differences in HIV/HCV prevalence according to differentiated risk behaviors in a sample of PWID in rural Puerto Rico. *Harm Reduction Journal* 2016; **13 (1) (no pagination)**(10).
374. Reitox National Focal Point. National Report on Drugs 2014. Romania: New Developments and Trends. 2015.
375. National Anti-Drug Agency, Reitox National Focal Point. 2013 National Report (2012 data) to the EMCDDA by the Reitox National Focal Point. Romania: New Developments, Trends and In-depth Information on Selected Issues, 2014.
376. Reitox National Focal Point, Oprea S, Iliescu R, et al. 2011 National Report (2010 data) to the EMCDDA by the Reitox National Focal Point. Romania: New Developments, Trends and In-depth Information on Selected Issues, 2012.
377. Reitox National Focal Point, Iliescu R, Lefter A, et al. 2012 National Report (2011 data) to the EMCDDA. Romania: New Developments, Trends and In-depth Information on Selected Issues, 2013.

378. UNODC. HIV, HBV and HCV Behavioral Surveillance Survey among Injecting Drug Users in Bucharest, Romania. 2011.
379. Sultana C, Vagu C, Temereanca A, Grancea C, Slobozeanu J, Ruta S. Hepatitis C virus genotypes in injecting drug users from Romania. *Central European Journal of Medicine* 2011; **6**(5): 672-8.
380. Platt L, Rhodes T, Hickman M, et al. Changes in HIV prevalence and risk among new injecting drug users in a Russian city of high HIV prevalence. *JAIDS Journal of Acquired Immune Deficiency Syndromes* 2008; **47**(5): 623-31.
381. Platt L, Sutton A, Vickerman P, et al. Measuring risk of HIV and HCV among injecting drug users in the Russian Federation. *European Journal of Public Health* 2009; **19**(4): 428-33.
382. Wall M, Schmidt E, Sarang A, Atun R, Renton A. Sex, drugs and economic behaviour in Russia: A study of socio-economic characteristics of high risk populations. *International Journal of Drug Policy* 2011; **22**(2): 133-9.
383. Cepeda JA, Odinokova VA, Heimer R, et al. Drug network characteristics and HIV risk among injection drug users in Russia: the roles of trust, size, and stability. *AIDS & Behavior* 2011; **15**(5): 1003-10.
384. Gyarmathy V, Li N, Tobin KE, et al. Correlates of unsafe equipment sharing among injecting drug users in St. Petersburg, Russia. *European Addiction Research* 2009; **15**(3): 163-70.
385. Heimer R, Barbour R, Shaboltas AV, Hoffman IF, Kozlov AP. Spatial distribution of HIV prevalence and incidence among injection drugs users in St Petersburg: Implications for HIV transmission. *Aids* 2008; **22**(1): 123-30.
386. Niccolai LM, Verevochkin SV, Toussova OV, et al. Estimates of HIV incidence among drug users in St. Petersburg, Russia: continued growth of a rapidly expanding epidemic. *European Journal of Public Health* 2011; **21**(5): 613-9.
387. Cepeda JA, Niccolai LM, Eritsyan K, Heimer R, Levina O. Moderate/heavy alcohol use and HCV infection among injection drug users in two Russian cities. *Drug & Alcohol Dependence* 2013; **132**(3): 571-9.
388. Eritsyan K, Heimer R, Barbour R, et al. Individual-level, network-level and city-level factors associated with HIV prevalence among people who inject drugs in eight Russian cities: A cross-sectional study. *BMJ Open* 2013; **3**(6): 1-11.
389. Abdala N, Krasnoselskikh TV, Durante AJ, Timofeeva MY, Verevochkin SV, Kozlov AP. Sexually transmitted infections, sexual risk behaviors and the risk of heterosexual spread of HIV among and beyond IDUs in St. Petersburg, Russia. *European Addiction Research* 2008; **14**(1): 19-25.

390. Alshomrani AT. Prevalence of human immunodeficiency virus, hepatitis C virus, and hepatitis B virus infection among heroin injectors in the central region of Saudi Arabia. *Saudi Medical Journal* 2015; **36**(7): 802-6.

391. Kimber J, Copeland L, Hickman M, et al. Survival and cessation in injecting drug users: prospective observational study of outcomes and effect of opiate substitution treatment. *BMJ* 2010: 8.

392. Munro A, Taylor A, Knox T, et al. Needle Exchange Surveillance Initiative (NESI): Prevalence of HCV and injecting risk behaviours among people who inject drugs (PWID) attending injecting equipment provision services (IEPs) in Scotland, 2008/2009-2013/2014. Scotland: University of the West of Scotland, 2015.

393. Matheson C, Anthony GB, Bond C, Rossi MK. Assessing and prioritizing the preferences of injecting drug users in needle and syringe exchange service development. *Journal of Public Health* 2008; **30**(2): 133-8.

394. Aspinall E, Hutchinson S, Taylor A, et al. Uptake of paraphernalia from injecting equipment provision services and its association with sharing of paraphernalia among injecting drug users in Scotland. *Drug and Alcohol Dependence* 2012; **126**(3): 340-6.

395. Coull AF, Atherton I, Taylor A, Watterson AE. Prevalence of skin problems and leg ulceration in a sample of young injecting drug users. *Harm Reduction Journal* 2014; **11**: 22.

396. O'Leary MC, Hutchinson SJ, Allen E, et al. The association between alcohol use and hepatitis C status among injecting drug users in Glasgow. *Drug & Alcohol Dependence* 2012; **123**(1-3): 180-9.

397. Stephens BP. Is it worth testing unstable drug users for hepatitis C? *Gut* 2011; **60**: A31-A2.

398. Kilibarda B, Simic D, Baros S, Ministry of Health, Brandic I. National Report on Drug Situation in Serbia, 2015.

399. National Institute of Public Health: Kosovo. HIV Integrated Behavioral and Biological Surveillance Surveys-Kosovo. 2014.

400. Bibi J, Faure J, Johnston L, Sinon F, Isnard R, Mangroo G. Injection Drug Use in the Republic of Seychelles, 2011. Integrated Biological and Behavioral Surveillance Survey - Round 1, 2011.

401. UNAIDS. Population Size Estimation of Key Populations. 2013.

402. Kinson RM, Guo S, Wan YM, Manning V, Teoh HC, Wong KE. Burden of blood transmitted infections in substance users admitted for inpatient treatment in Singapore and the associated factors. *Singapore Medical Journal* 2015; **56**(2): 87-91.

403. Gazdikova K, Gazdik F, Kajaba I, Huckova D, Okruhlica L, Farkasova D. The seroprevalence of HCV among injecting drug users in the years 2004-2008 in Slovakia. *Vnitrni Lekarstvi* 2012; **58**(3): 179-82.

404. Reitox National Focal Point, Drev A, Stokelj R, et al. 2012 National Report (2011 data) to the EMCDDA. Slovenia: New developments, trends and in-depth information on selected issues. 2013.
405. Pares-Badell O, Espelt A, Folch C, et al. Undiagnosed HIV and Hepatitis C infection in people who inject drugs: From new evidence to better practice. *Journal of Substance Abuse Treatment* 2017; **77**: 13-20.
406. Reitox National Focal Point. 2010 National Report (2009 data) to the EMCDDA. Spain: New Development, Trends and in-depth information on selected issues, 2011.
407. Reitox National Focal Point, Ministerio de Sanidad de Espania. 2012 National Report (2011 data) to the EMCDDA. Spain: New Development, Trends and in-depth information on selected issues, 2013.
408. Reitox National Focal Point. 2011 National Report (2010 data) to the EMCDDA. Spain: New developments, trends and in-depth information on selected issues, 2012.
409. Sanvisens A, Bolao F, Vallecillo G, et al. HIV Infection and Viral Hepatitis in Drug Abusers. In: Saxena SK, ed. Current Perspectives in HIV Infection: INTECH; 2013: 367-84.
410. Reitox National Focal Point. 2013 National Report (2012 data) for the EMCDDA. "Spain" New Development, Trends and In-depth Information on Selected Issues.: Ministerio de Sanidad, Servicios Sociales e Igualdad, 2014.
411. Saigi N, Espelt A, Folch C, et al. Differences in illegal drug consumption between native and immigrants in a large sample of injected drug users in Catalonia (Spain). *Adicciones* 2014; **26**(1): 69-76.
412. Sarasa-Renedo A, Espelt A, Folch C, et al. Overdose prevention in injecting opioid users: the role of substance abuse treatment and training programs. *Gaceta Sanitaria* 2014; **28**(2): 146-54.
413. Diez M, Bleda MJ, Varela JR, et al. Trends in HIV testing, prevalence among first-time testers, and incidence in most-at-risk populations in Spain: the EPI-VIH Study, 2000 to 2009. *Euro Surveillance: Bulletin Europeen sur les Maladies Transmissibles = European Communicable Disease Bulletin* 2014; **19**(47): 20971.
414. Folch C, Casabona J, Espelt A, et al. Gender differences in HIV risk behaviours among intravenous drug users in Catalonia, Spain. *Gaceta Sanitaria* 2013; **27**(4): 338-43.
415. Rivas I, Martinez E, Sanvisens A, et al. Hepatitis B virus serum profiles in injection drug users and rates of immunization over time in Barcelona: 1987-2006. *Drug & Alcohol Dependence* 2010; **110**(3): 234-9.
416. Huntington S, Folch C, Gonzalez V, Merono M, Ncube F, Casabona J. Prevalence of human immunodeficiency virus and hepatitis C virus, and associated factors among injecting drug users in Catalonia. [Spanish] Prevalencia del VIH, hepatitis C y factores asociados en usuarios de drogas por via parenteral seleccionados en Cataluna. *Enfermedades Infecciosas y Microbiologia Clinica* 2010; **28**(4): 236-8.

417. Hurtado Navarro I, Alastrue I, Del Amo J, et al. Differences between women and men in serial HIV prevalence and incidence trends. *European Journal of Epidemiology* 2008; **23**(6): 435-40.
418. Global Fund. IBBS Survey in Sri lanka 2015.
419. Dahlman D, Jalalvand F, Blome MA, et al. High Perineal and Overall Frequency of Staphylococcus aureus in People Who Inject Drugs, Compared to Non-Injectors. *Current Microbiology* 2017; **74**(2): 159-67.
420. Dahlman D, Hakansson A, Bjorkman P, Blome MA, Kral AH. Correlates of skin and soft tissue infections in injection drug users in a syringe-exchange program in Malmo, Sweden. *Substance Use & Misuse* 2015; **50**(12): 1529-35.
421. Hakansson A, Isendahl P, Wallin C, Berglund M. Efficacy of mobile telephone contact for follow-up in injecting heroin users. *American Journal of Drug & Alcohol Abuse* 2011; **37**(2): 89-92.
422. Hakansson A, Isendahl P, Wallin C, Berglund M. Respondent-driven sampling in a syringe exchange setting. *Scandinavian Journal of Public Health* 2012; **40**(8): 725-9.
423. Blome MA, Bjorkman P, Flamholc L, Jacobsson H, Molnegren V, Widell A. Minimal transmission of HIV despite persistently high transmission of hepatitis C virus in a Swedish needle exchange program. *Journal of Viral Hepatitis* 2011; **18**(12): 831-9.
424. Jerkeman A, Westin J, Lagging M, et al. Chronic hepatitis C in Swedish subjects receiving opiate substitution therapy-Factors associated with advanced fibrosis. *Scandinavian Journal of Infectious Diseases* 2014; **46**(5): 340-7.
425. Hillgren K, Sarkar K, Elofsson S, Britton S. Widespread risk behavior among injecting drug users. *Lakartidningen* 2012; **109**(25): 1221-5.
426. Dickson-Spillmann M, Haug S, Uchtenhagen A, Bruggmann P, Schaub MP. Rates of HIV and Hepatitis Infections in Clients Entering Heroin-Assisted Treatment between 2003 and 2013 and Risk Factors for Hepatitis C Infection. *European Addiction Research* 2016; **22**(4): 181-91.
427. UNDP. Syria IBBS. 2014.
428. Lee TSH, Shen HC, Wu WH, et al. Clinical characteristics and risk behavior as a function of HIV status among heroin users enrolled in methadone treatment in northern Taiwan. *Substance Abuse Treatment, Prevention, & Policy* 2011; **6**: 6.
429. Yen YF, Hu BS, Lin YS, et al. Latent tuberculosis among injection drug users in a methadone maintenance treatment program, Taipei, Taiwan: TSPOT.TB versus tuberculin skin test. *Scandinavian Journal of Infectious Diseases* 2013; **45**(7): 504-11.
430. Fu TST, Tuan YC, Yen MY, et al. Psychometric properties of the World Health Organization quality of life assessment-Brief in methadone patients: A validation study in northern Taiwan. *Harm Reduction Journal* 2013; **10**: 37.

431. Yen YF, Rodwell TC, Yen MY, et al. HIV infection risk among injection drug users in a methadone maintenance treatment program, Taipei, Taiwan 20072010. *American Journal of Drug and Alcohol Abuse* 2012; **38**(6): 544-50.

432. Yen YF, Yen MY, Lin T, et al. Prevalence and factors associated with HIV infection among injection drug users at methadone clinics in Taipei, Taiwan. *BMC Public Health* 2014; **14**: 682.

433. Ko NY, Wang PW, Wu HC, et al. Self-efficacy and HIV risk behaviors among heroin users in Taiwan. *Journal of Studies on Alcohol & Drugs* 2012; **73**(3): 469-76.

434. Bangel SA, Zule W, Otiashvili D, Latypov A, Wechsberg M. Gender disparities in HIV prevalence and risk behaviors among people who inject drugs in Tajikistan. 2016.

435. Beyrer C, Patel Z, Stachowiak JA, et al. Characterization of the emerging HIV type 1 and HCV epidemics among injecting drug users in Dushanbe, Tajikistan. *AIDS Research & Human Retroviruses* 2009; **25**(9): 853-60.

436. National AIDS and Control Programme (NACP), Muhimnili University of Health and Allied Sciences. Integrated Bio-Behavioral Survey Among People Who Inject Drugs in Dar es Salaam, Tanzania, 2014: Muhimnili University of Health and Allied Sciences,, 2014.

437. Mlunde LB, Sunguya BF, Mbwambo JK, et al. A mismatch between high-risk behaviors and screening of infectious diseases among people who inject drugs in Dar es Salaam, Tanzania. *PloS one* 2016; **11 (2) (no pagination)**(e0148598).

438. Tan AX, Kapiga S, Khoshnood K, Bruce R. Epidemiology of drug use and HIV-related risk behaviors among people who inject drugs in Mwanza, Tanzania. *PloS one* 2015; **10**(12).

439. Gupta A, Mbwambo J, Mteza I, et al. Active case finding for tuberculosis among people who inject drugs on methadone treatment in Dar es Salaam, Tanzania. *International Journal of Tuberculosis and Lung Disease* 2014; **18**(7): 793-8+i.

440. Lambdin BH, Bruce RD, Chang O, et al. Identifying Programmatic Gaps: Inequities in Harm Reduction Service Utilization among Male and Female Drug Users in Dar es Salaam, Tanzania. *PloS one* 2013; **8 (6) (no pagination)**(e67062).

441. Lambdin BH, Masao F, Chang O, et al. Methadone treatment for HIV prevention- Feasibility, retention, and predictors of attrition in Dar es Salaam, Tanzania: A Retrospective cohort study. *Clinical Infectious Diseases* 2014; **59**(5): 735-42.

442. Ross MW, McCurdy SA, Kilonzo GP, Williams ML, Leshabari MT. Drug use careers and blood-borne pathogen risk behavior in male and female Tanzanian heroin injectors. *American Journal of Tropical Medicine and Hygiene* 2008; **79**(3): 338-43.

443. Matiko E, Khatib A, Khalid F, et al. HIV prevalence and risk behaviors among people who inject drugs in two serial cross-sectional respondent-driven sampling surveys, Zanzibar 2007 and 2012. *AIDS & Behavior* 2015; **19 Suppl 1**: S36-45.
444. Prybylski D, Manopaiboon C, Visavakum P, et al. Diverse HIV epidemics among people who inject drugs in Thailand: evidence from respondent-driven sampling surveys in Bangkok and Chiang Mai. *Drug & Alcohol Dependence* 2015; **148**: 126-35.
445. Pansuwan N, Wisawakam P, Saengwanloy O, Jittakot Y, Pawa D. The 2012 Integrated Behavior and Biological Surveillance (IBBS) of HIV, Sexually Transmitted Infections and Associated Risk Behaviors among Injecting Drug Users. 2012.
446. Visavakum P, Punsuwan N, Manopaiboon C, et al. HIV prevalence and risk behaviors among people who inject drugs in Songkhla, Thailand: A respondent-driven sampling survey. *International Journal of Drug Policy* 2016; **31**: 163-7.
447. Fairbairn N, Hayashi K, Kaplan K, et al. Factors associated with methadone treatment among injection drug users in Bangkok, Thailand. *Journal of Substance Abuse Treatment* 2012; **43**(1): 108-13.
448. Fairbairn N, Hayashi K, Ti L, et al. Compulsory drug detention and injection drug use cessation and relapse in Bangkok, Thailand. *Drug and Alcohol Review* 2015; **34**(1): 74-81.
449. Hayashi K, Wood E, Suwannawong P, Kaplan K, Qi J, Kerr T. Methamphetamine injection and syringe sharing among a community-recruited sample of injection drug users in Bangkok, Thailand. *Drug and Alcohol Dependence* 2011; **115**(1-2): 145-9.
450. Kerr T, Kiatying-Angsulee N, Fairbairn N, et al. High rates of midazolam injection among drug users in Bangkok, Thailand. *Harm Reduction Journal* 2010; **7**: 7.
451. Sunthornchart S, Linkins RW, Natephisarnwanish V, et al. Prevalence of hepatitis B, tetanus, hepatitis A, human immunodeficiency virus and feasibility of vaccine delivery among injecting drug users in Bangkok, Thailand, 2003-2005. *Addiction* 2008; **103**(10): 1687-95.
452. UNAIDS. Cartographie des sites, estimation de la taille des usagers de drogues injectables (UDI) et evaluation de leurs besoins en matiere de services deprevention, de soins traitement en matiere de VIH et de sante de la reproduction au Togo. 2014.
453. Benzineb S, Hsairi M. Enquête de séroprévalence du VIH et des Hépatites virales auprès des usagers de drogues injectables en Tunisie. 2009.
454. Tunisia Ministry of Health, Tunisian Association for Information and Orientation on HIV. Enquête sérocomportementale du VIH et des hépatites virales C auprès des usagers de drogues injectables en Tunisie. 2015.
455. Reitox National Focal Point, Tasdemir A, Kocak N, et al. 2011 National Report (2010 data) to the EMCDDA. "Turkey" New Development, Trends and in-depth information on selected issues, 2012.

456. Alaei A, Alaei K, Waye K, et al. Hepatitis C infection and other drug-related harms among inpatients who injected drugs in Turkey. *Journal of Viral Hepatitis* 2016.

457. Kutsa O, Marcus R, Bojko MJ, et al. Factors associated with physical and sexual violence by police among people who inject drugs in Ukraine: Implications for retention on opioid agonist therapy. *Journal of the International AIDS Society* 2016; **19 (no pagination)**(20897).

458. Booth RE, Davis JM, Brewster JT, Lisovska O, Dvoryak S. Krokodile injectors in Ukraine: Fueling the HIV epidemic? *AIDS and Behavior* 2016; **20**(2): 369-76.

459. International HIV/AIDS Alliance in Ukraine. Monitoring the Behavior of Injecting Drug Users. 2008.

460. Balakiryeva OM, Bondar TV, Sereda YV, Sazonova YO. Behavior Monitoring and HIV Prevalence among Injecting Drug Users as a Component of Second Generation Sentinell Surveillance. 2012.

461. Global Fund. МОНІТОРИНГ ПОВЕДІНКИ ТА ПОШИРЕННЯ ВІЛ-ІНФЕКЦІЇ СЕРЕД СПОЖИВАЧІВ ІН'ЄКЦІЙНИХ НАРКОТИКІВ ЯК КОМПОНЕНТ ЕПІДНАГЛЯДУ ЗА ВІЛ ДРУГОГО ПОКОЛІННЯ. 2014.

462. Pohorila N, Taran Y, Kolodiy I, Diyeva T. Behavior monitoring and HIV-infection prevalence among injection drug users. 2009.

463. Barska G, Sazonov JO. Survey Results 2015. Monitoring Behaviour and HIV Prevalence among People who use Injectable Drugs and their Sexual Partners, 2016.

464. Booth RE, Lehman WEK, Dvoryak S, Brewster JT, Sinitsyna L. Interventions with injection drug users in Ukraine. *Addiction* 2009; **104**(11): 1864-73.

465. Dumchev KV, Soldyshev R, Qian HZ, et al. HIV and hepatitis C virus infections among hanka injection drug users in central Ukraine: A cross-sectional survey. *Harm Reduction Journal* 2009; **6**: 23.

466. Schaub M, Chtenguelov V, Subata E, Weiler G, Uchtenhagen A. Feasibility of buprenorphine and methadone maintenance programmes among users of home made opioids in Ukraine. *International Journal of Drug Policy* 2010; **21**(3): 229-33.

467. Behrends CN. Evaluating the Impact of Satellite Syringe Exchange on Reducing HIV Risk Behavior and Seroconversion among People who Inject Drugs. ProQuest LLC: University of California Davis; 2014.

468. Grau LE, Zhan W, Heimer R. Prevention knowledge, risk behaviours and seroprevalence among nonurban injectors of southwest Connecticut. *Drug and Alcohol Review* 2016; **35**(5): 628-36.

469. Ruiz MS, O'Rourke A, Allen ST. Using Capture-Recapture Methods to Estimate the Population of People Who Inject Drugs in Washington, DC. *AIDS and behavior* 2016; **20**(2): 363-8.

470. Summers PJ, Struve IA, Wilkes MS, Rees VW. Injection-site vein loss and soft tissue abscesses associated with black tar heroin injection: A cross-sectional study of two distinct populations in USA. *International Journal of Drug Policy* 2017; **39**: 21-7.
471. Armenta RF, Collins KM, Strathdee SA, et al. Mycobacterium tuberculosis infection among persons who inject drugs in San Diego, California. *International Journal of Tuberculosis and Lung Disease* 2017; **21**(4): 425-31.
472. Des Jarlais DC, Arasteh K, McKnight C, et al. Providing ART to HIV seropositive persons who use drugs: Progress in New York City, prospects for "ending the epidemic". *AIDS and Behavior* 2016; **20**(2): 353-62.
473. Zeremski M, Dimova RB, Zavala R, et al. Hepatitis C Virus-Related Knowledge and Willlingness to Receive Treatment Among Patients on Methadone Maintenance. *Journal of Addiction Medicine* 2014; **8**(4): 249-57.
474. Centers for Disease Control and Prevention. HIV Infection, Risk, Prevention, and Testing Behaviors among Persons Who Inject Drugs: National HIV Behavioral Surveillance Injection Drug Use 20 U.S. Cities, 2012. 2015.
475. Schmitz J, Kral AH, Chu D, Wenger L, Bluthenthal R. Food insecurity among people who inject drugs in Los Angeles and San Francisco. *Public Health Nutrition* 2016: 1-9.
476. Hwang L, Grimes CZ. Human Immunodeficiency Virus, Hepatitis B and Hepatitis C Virus Infections Among Injecting and Non-Injecting Drug Users in Inner City Neighborhoods; 2012.
477. Mino M, Deren S, Colon HM. HIV and drug use in Puerto Rico: Findings from the ARIBBA study. *Journal of the International Association of Physicians in AIDS Care* 2011; **10**(4): 248-59.
478. Quinn B, Chu D, Wenger L, Bluthenthal RN, Kral AH. Syringe disposal among people who inject drugs in Los Angeles: The role of sterile syringe source. *International Journal of Drug Policy* 2014; **25**(5): 905-10.
479. Sirikantraporn S, Mateu-Gelabert P, Friedman SR, Sandoval M, Torruella RA. Resilience among IDUs: Planning strategies to help injection drug users to protect themselves and others from HIV/HCV infections. *Substance Use & Misuse* 2012; **47**(10): 1125-33.
480. Stein M, Thurmond P, Bailey G. Willingness to use HIV pre-exposure prophylaxis among opiate users. *AIDS & Behavior* 2014; **18**(9): 1694-700.
481. Stopka TJ, Lutnick A, Wenger LD, DeRiemer K, Geraghty EM, Kral AH. Demographic, risk, and spatial factors associated with over-the-counter syringe purchase among injection drug users. *American Journal of Epidemiology* 2012; **176**(1): 14-23.

482. Tookes HE, Kral AH, Wenger LD, et al. A comparison of syringe disposal practices among injection drug users in a city with versus a city without needle and syringe programs. *Drug and Alcohol Dependence* 2012; **123**(1-3): 255-9.
483. Zaller ND, Yokell MA, Nayak SM, Fu JJ, Bazazi AR, Rich JD. Syringe acquisition experiences and attitudes among injection drug users undergoing short-term opioid detoxification in Massachusetts and Rhode Island. *Journal of Urban Health* 2012; **89**(4): 659-70.
484. Zulea WA, Bobashev G. High dead-space syringes and the risk of HIV and HCV infection among injecting drug users. *Drug and Alcohol Dependence* 2009; **100**(3): 204-13.
485. Lopez WD, Krueger PM, Walters ST. High-risk drug use and sexual behaviors among out-of-treatment drug users: an aging and life course perspective. *Addictive Behaviors* 2010; **35**(5): 432-7.
486. Martinez AN, Bluthenthal RN, Neilands T, Kral AH. Assessing geographic and individual level factors associated with arrests among injection drug users in California. *Health and Place* 2011; **17**(6): 1258-65.
487. Amodeo M, Lundgren L, Chassler D, Witas J. High-frequency users of detoxification: who are they? *Substance Use & Misuse* 2008; **43**(7): 839-49.
488. Barocas JA, Baker L, Hull SJ, Stokes S, Westergaard RP. High uptake of naloxone-based overdose prevention training among previously incarcerated syringe-exchange program participants. *Drug and Alcohol Dependence* 2015; **154**: 283-6.
489. Bazazi AR, Yokell M, Fu JJ, Rich JD, Zaller ND. Illicit use of buprenorphine/naloxone among injecting and noninjecting opioid users. *Journal of Addiction Medicine* 2011; **5**(3): 175-80.
490. Beletsky L, Cochrane J, Sawyer AL, et al. Police encounters among needle exchange clients in Baltimore: Drug law enforcement as a structural determinant of health. *American Journal of Public Health* 2015; **105**(9): 1872-9.
491. Cisneros GO, Douaihy AB, Kirisci L. Access to healthcare among injection drug users at a needle exchange program in Pittsburgh, PA. *Journal of Addiction Medicine* 2009; **3**(2): 89-94.
492. Coffin PO, Coffin LS, Murphy S, Jenkins LM, Golden MR. Prevalence and characteristics of femoral injection among Seattle-area injection drug users. *Journal of Urban Health* 2012; **89**(2): 365-72.
493. Copersino ML, Meade CS, Bigelow GE, Brooner RK. Measurement of self-reported HIV risk behaviors in injection drug users: Comparison of standard versus timeline follow-back administration procedures. *Journal of Substance Abuse Treatment* 2010; **38**(1): 60-5.
494. Corsi KF, Kwiatkowski CF, Booth RE. Predictors of methamphetamine injection in out-of-treatment IDUs. *Substance Use & Misuse* 2009; **44**(3): 332-42.

495. Friedman SR, Pouget ER, Sandoval M, Jones Y, Mateu-Gelabert P. Formal and informal organizational activities of people who inject drugs in New York City: description and correlates. *Journal of Addictive Diseases* 2015; **34**(1): 55-62.
496. Gindi RM, Rucker MG, Serio-Chapman CE, Sherman SG. Utilization patterns and correlates of retention among clients of the needle exchange program in Baltimore, Maryland. *Drug and Alcohol Dependence* 2009; **103**(3): 93-8.
497. Heller DI, Paone D, Siegler A, Karpati A. The syringe gap: An assessment of sterile syringe need and acquisition among syringe exchange program participants in New York City. *Harm Reduction Journal* 2009; **6**: 1.
498. Huo D, Ouellet LJ. Needle exchange and sexual risk behaviors among a cohort of injection drug users in Chicago, Illinois. *Sexually Transmitted Diseases* 2009; **36**(1): 35-40.
499. Jenkins LM, Banta-Green CJ, Maynard C, et al. Risk factors for nonfatal overdose at Seattle-area syringe exchanges. *Journal of Urban Health* 2011; **88**(1): 118-28.
500. Zibbell JE, Hart-Malloy R, Barry J, Fan L, Flanigan C. Risk factors for HCV infection among young adults in rural New York who inject prescription opioid analgesics. *American Journal of Public Health* 2014; **104**(11): 2226-32.
501. Havens JR, Lofwall MR, Frost SDW, Oser CB, Leukefeld CG, Crosby RA. Individual and network factors associated with prevalent hepatitis C infection among rural Appalachian injection drug users. *American Journal of Public Health* 2013; **103**(1): e44-52.
502. Jordan AE, Des Jarlais DC, Arasteh K, McKnight C, Nash D, Perlman DC. Incidence and prevalence of hepatitis c virus infection among persons who inject drugs in New York City: 2006-2013. *Drug & Alcohol Dependence* 2015; **152**: 194-200.
503. Mehta SH, Astemborski J, Kirk GD, et al. Changes in blood-borne infection risk among injection drug users. *Journal of Infectious Diseases* 2011; **203**(5): 587-94.
504. Oster AM, Sternberg M, Nebenzahl S, et al. Prevalence of HIV, sexually transmitted infections, and viral hepatitis by Urbanicity, among men who have sex with men, injection drug users, and heterosexuals in the United States. *Sexually Transmitted Diseases* 2014; **41**(4): 272-9.
505. Neaigus A, Zhao M, Gyarmathy VA, Cisek L, Friedman SR, Baxter RC. Greater drug injecting risk for HIV, HBV, and HCV infection in a city where syringe exchange and pharmacy syringe distribution are illegal. *Journal of Urban Health* 2008; **85**(3): 309-22.
506. Ministry of Health. HIV/STI Integrated Biological and Behavioral Surveillance (IBBS) in Vietnam. Results from Round III 2013 and Trends Across Three Rounds (2005-2009-2013) of Surveys. 2014.

507. Des Jarlais D, Huong DT, Oanh KTH, et al. Prospects for ending the HIV epidemic among persons who inject drugs in Haiphong, Vietnam. *International Journal of Drug Policy* 2016; **32**.

508. Nguyen CH, Ishizaki A, Chung PTT, et al. Prevalence of HBV infection among different HIV-risk groups in Hai Phong, Vietnam. *Journal of Medical Virology* 2011; **83**(3): 399-404.

509. Tanimoto T, Nguyen HC, Ishizaki A, et al. Multiple routes of hepatitis C virus transmission among injection drug users in Hai Phong, Northern Vietnam. *Journal of Medical Virology* 2010; **82**(8): 1355-63.

510. Zhang L, Celentano DD, Minh NL, et al. Prevalence and correlates of HCV monoinfection and HIV and HCV coinfection among persons who inject drugs in Vietnam. *European Journal of Gastroenterology and Hepatology* 2015; **27**(5): 550-6.

511. Khuat OT, Morrow M, Nguyen TN, Armstrong G. Social context, diversity and risk among women who inject drugs in Vietnam: Descriptive findings from a cross-sectional survey. *Harm Reduction Journal* 2015; **12**: 35.

512. Lim TW, Frangakis C, Latkin C, et al. Community-level income inequality and HIV prevalence among persons who inject drugs in Thai Nguyen, Vietnam. *PLoS ONE [Electronic Resource]* 2014; **9**(3): e90723.

513. Nadol P, O'Connor S, Duong H, et al. Findings from integrated behavioral and biologic survey among males who inject drugs (MWID) - Vietnam, 2009-2010: evidence of the need for an integrated response to HIV, hepatitis B virus, and hepatitis C virus. *PLoS ONE [Electronic Resource]* 2015; **10**(2): e0118304.

514. Bergenstrom A, Quan VM, Van Nam L, et al. A cross-sectional study on prevalence of non-fatal drug overdose and associated risk characteristics among out-of-treatment injecting drug users in North Vietnam. *Substance Use & Misuse* 2008; **43**(1): 73-84.

515. Craine N, Hickman M, Parry JV, Smith J, McDonald T, Lyons M. Characteristics of injecting drug users accessing different types of needle and syringe programme or using secondary distribution. *Journal of Public Health* 2010; **32**(3): 328-35.

Supplementary materials: Sensitivity analyses

Sensitivity analyses were performed for the analyses with the percentage of young PWID as the outcome and for calculating the global percentage of PWID that are young: a) defining young PWID as those aged 30 years or under, rather than 25 years or under; and b) excluding Nepal, Estonia, and the Philippines from the analysis as the estimate for these three countries incorporated studies where the youngest age group was either 16-19, 15-17, or 18-20 years.

The sensitivity analyses for the regression with the percentage of PWID that are young as the outcome a) defining young PWID as aged under 30, and b) excluding Nepal, Estonia, and the Philippines, were both similar to the main analysis, see Supplementary table 3. The global percentage of PWID that are young for sensitivity analysis a) is 26.9% (95% CI: 19.2%, 35.6%), whilst for sensitivity analysis b) it is 25.0% (95% CI: 19.5%, 31.5%).

Supplementary table 1: The number and percentage of studies using each youngest age group category.

Youngest age category	N (%) of studies	Only included in sensitivity
18-24	87 (23.84%)	No
<25	36 (9.86%)	No
15-24	32 (8.77%)	No
18-25	28 (7.67%)	No
18-29	20 (5.48%)	Yes
16-24	18 (4.93%)	No
<31	18 (4.93%)	Yes
18-30	17 (4.66%)	Yes
<26	14 (3.84%)	No
15-25	11 (3.01%)	No
<30	11 (3.01%)	Yes
16-19	7 (1.92%)	No
16-25	6 (1.64%)	No
16-29	6 (1.64%)	Yes
15-29	5 (1.37%)	Yes
17-29	5 (1.37%)	Yes
18-26	5 (1.37%)	Yes
14-24	4 (1.1%)	No
18-28	3 (0.82%)	Yes
15-30	2 (0.55%)	Yes
17-25	2 (0.55%)	No
18-20	2 (0.55%)	No
19-29	2 (0.55%)	Yes
20-24	2 (0.55%)	No
20-25	2 (0.55%)	No
20-29	2 (0.55%)	Yes
20-30	2 (0.55%)	Yes
10-25	1 (0.27%)	No
11-29	1 (0.27%)	Yes
13-24	1 (0.27%)	No
14-29	1 (0.27%)	Yes
15-17	1 (0.27%)	No
15-27	1 (0.27%)	Yes
17-24	1 (0.27%)	No
19-24	1 (0.27%)	No
19-25	1 (0.27%)	No
21-24	1 (0.27%)	No
21-30	1 (0.27%)	Yes
22-30	1 (0.27%)	Yes
23-30	1 (0.27%)	Yes
<24	1 (0.27%)	No
<27	1 (0.27%)	Yes

Supplementary table 2: Sensitivity analyses for linear regressions of country-level predictors of the % of PWID that are young (logit transformed); a) defining young PWID as those aged 30 or under; and b) excluding Nepal, Philippines, and Estonia – the countries that include studies (N=10) where the youngest age group is either 15-17, 16-19, or 18-20.

	**A: Defining young PWID as those aged under 30 (N = 68)**				
	Univariable			Multivariable (N=NA)	
	N	β (95% CI)	p	β (95% CI)	p
Urban population growth	68	-0.02 (-0.13, 0.09)	0.727		
% Youth unemployment	67	0.00 (-0.02, 0.02)	0.984		
GDP (per $1000 increase)	66	0.00 (-0.01, 0.01)	0.978		
GINI (per score increase)	66	-0.01 (-0.04, 0.02)	0.553		
OST coverage (per % increase)	57	0.00 (-0.01, 0.01)	0.792		
% Female PWID	60	0.00 (-0.02, 0.02)	0.677		
% Gen-pop aged 15-24	65	-0.01 (-0.07, 0.05)	0.771		
	**B) Excluding Nepal, Philippines, and Estonia (N = 57)**				
	Univariable			Multivariable (N=NA)	
	N	β (95% CI)	p	β (95% CI)	p
Urban population growth	57	-0.03 (-0.14, 0.08)	0.563		
% Youth unemployment	57	0.00 (-0.01, 0.02)	0.724		
GDP (per $1000 increase)	56	-0.01 (-0.02, 0.01)	0.391		
GINI (per score increase)	56	0.00 (-0.03, 0.03)	0.954		
OST coverage (per % increase)	48	-0.00 (-0.01, 0.00)	0.188		
% Female PWID	50	0.00 (-0.02, 0.03)	0.685		
% Gen-pop aged 15-24	54	-0.00 (-0.06, 0.05)	0.875		
